# Automated scoring of airway abnormalities and mucus plugging in chest magnetic resonance imaging of cystic fibrosis using artificial intelligence

**DOI:** 10.1016/j.csbj.2025.10.025

**Published:** 2025-10-15

**Authors:** Friedemann G. Ringwald, Lena Wucherpfennig, Anna Martynova, Niclas Hagen, Jacqueline Kürschner, Shengkai Zhao, Mirjam Stahl, Olaf Sommerburg, Marcus A. Mall, Simon Y. Graeber, Eva Steinke, Petra Knaup, Mark O. Wielpütz, Urs Eisenmann

**Affiliations:** a Institute of Medical Informatics, Heidelberg University, Im Neuenheimer Feld 130.3, Heidelberg 69120, Germany; b Translational Lung Research Center Heidelberg (TLRC), German Center for Lung Research (DZL), Im Neuenheimer Feld 130.3, Heidelberg 69120, Germany; c Department of Diagnostic and Interventional Radiology, Heidelberg University Hospital, Im Neuenheimer Feld 420, Heidelberg 69120, Germany; d Department of Diagnostic and Interventional Radiology with Nuclear Medicine, Thoraxklinik at University Hospital Heidelberg, Röntgenstr. 1, Heidelberg 69126, Germany; e Department of Pediatric Respiratory Medicine, Immunology and Critical Care Medicine, Charité-Universitätsmedizin Berlin, Augustenburger Platz 1, Berlin 13353, Germany; f German Center for Lung Research (DZL), associated partner site Berlin, Augustenburger Platz 1, Berlin 13353, Germany; g German Center for Child and Adolescent Health (DZKJ) partner site Berlin, Augustenburger Platz 1, Berlin 13353, Germany; h Berlin Institute of Health (BIH) at Charité-Universitätsmedizin Berlin, Anna-Louisa-Karsch-Straße 2, Berlin 10178, Germany; i Division of Pediatric Pulmonology & Allergy and Cystic Fibrosis Center, Department of Pediatrics, University Hospital Heidelberg, Im Neuenheimer Feld 672, Heidelberg 69120, Germany; j Department of Translational Pulmonology, University Hospital Heidelberg, Im Neuenheimer Feld 672, Heidelberg 69120, Germany; k Department of Diagnostic Radiology and Neuroradiology, University Medicine Greifswald, Ferdinand-Sauerbruch-Straße 1, Greifswald 17475, Germany; l Department of Nuclear Medicine, University Medicine Greifswald, Ferdinand-Sauerbruch-Straße 1, Greifswald 17475, Germany; m Cluster of Excellence ImmunoPreCept, Charité – Universitätsmedizin Berlin, Berlin, Germany

**Keywords:** Cystic fibrosis, Deep learning, Magnetic resonance imaging, Lung

## Abstract

Cystic fibrosis is characterized by progressive lung damage, requiring life-long medical treatment and monitoring. This emphasizes the need for reliable, radiation-free imaging and automated analysis of lung disease activity. We present a deep learning-based approach for classifying two key pathologies, bronchiectasis/wall thickening and mucus plugging, on T2-weighted chest MRI. Retrospectively, 627 MRI scans from 164 patients (mean age 7.0 ± 6.2 years; range 0.1–53.0 years) were collected. Chest MRI were preprocessed with an nnU-Net to segment lung halves, followed by an atlas-based lung lobe approximation. Leveraging a dual-view architecture processing coronal and axial slices, our approach addresses limitations inherent in manual scoring, such as reader variability and substantial labor requirements. We evaluated a single model trained on all lobes and models specialized for each lobe. Cross-validation revealed substantial agreement for mucus plugging (κ = 0.68) with strong discrimination (macro AUROC = 0.90) and excellent reliability (Pearson’s r = 0.84). For bronchiectasis/wall thickening, agreement was moderate (κ = 0.53) but discrimination remained strong (macro AUROC = 0.87), with Pearson’s r = 0.74. The mean differences and 95 % limits of agreement for both pathologies aligned closely with the reader variability previously reported. Grad-CAM analyses demonstrated spatial alignment of model attention with relevant pathologies, and external testing on ten patients from an independent centre confirmed promising generalization. These findings represent a significant step toward automated MRI-based assessment for CF-related lung changes. Extending the approach to additional MRI scoring items may also improve granularity and clinical applicability, ultimately aiding in more personalized CF management.

## Graphical Abstract

**Figure:**
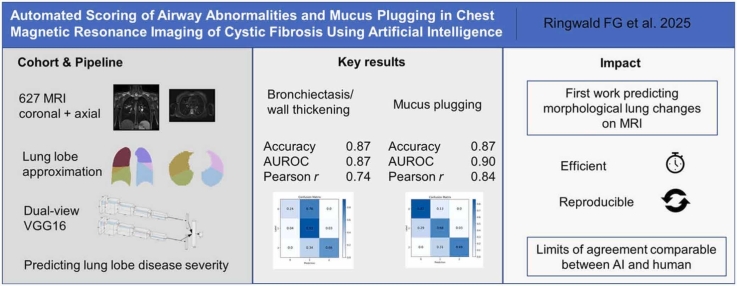

## 1 Introduction

Cystic fibrosis (CF) is characterized by progressive lung damage from birth, requiring life-long treatment and surveillance [1], [2], [3]. Recently, cystic fibrosis transmembrane conductance regulator (CFTR) modulators have provided effective treatment for CF patients, extending life expectancy and reducing morbidity. As individuals live longer with CF, new challenges have emerged, including the need for reliable and objective methods to quantify lung disease activity over time [4], [5], [6]. Magnetic resonance imaging (MRI), a radiation-free imaging modality, offers the ability to perform repeated measurements to assess morphological lung abnormalities from infancy to adulthood [7], [8], [9], [10].

With the increasing use of MRI for lung assessment, a semi-quantitative scoring system – the Eichinger Score – was developed and subsequently used in clinical trials [11], [12], [13], [14], [10], [15]. It enables a lobar-based evaluation of morphological and perfusion abnormalities. Automating the Eichinger Score holds significant potential for streamlining the clinical workflow, saving time, and providing consistent outputs that can support reliable and repeatable assessments.

In this context, artificial intelligence (AI) offers a powerful opportunity: leveraging advanced machine learning and deep learning algorithms, automated systems can learn from annotated datasets, thereby facilitating consistent, rapid, and reproducible evaluations [16], [17]. AI-driven methods can handle subtle variations in MR imaging protocols and diverse patient presentations, potentially improving early detection of CF-related lung alterations [18], [19]. By targeting individual components of the Eichinger Score – such as bronchiectasis/wall thickening and mucus plugging – automated classification systems may generate quantifiable metrics that align closely with established scoring criteria [20], [21], [22]. This can facilitate not only robust cross-sectional assessments but also the tracking of disease progression and therapeutic response over time. Ultimately, integrating AI into CF imaging analysis represents a pivotal step towards reducing reader subjectivity, streamlining the entire diagnostic process and providing timely, objective information that could optimize personalized treatment strategies [23].

To date, automated detection and classification of pulmonary changes has predominantly been performed on CT [18]. Advances in fully automated segmentation of lung parenchyma, lobes, and airways on CT have paved the way for detailed quantitative analyses of the morphological changes induced by a wide range of pulmonary diseases [24], [25]. Several studies have shown the potential of deep learning for identifying structural abnormalities and predicting clinical outcomes on CT. For instance, Sun et al. presented a weakly supervised model for detecting COPD, while Walsh et al. used deep learning to predict survival in fibrotic lung disease, surpassing conventional clinical and radiological predictors [26], [27]. In CF, Dournes et al. employed 2D convolutional networks to quantify CF-related changes, achieving close agreement with expert annotations and near-perfect reproducibility [28].

Building on these foundations, subsequent studies have specifically targeted bronchiectasis/wall thickening and mucus plugging: Aliboni et al. predicted bronchiectasis from cropped CT slices with high accuracies [29]. Yue et al. achieved high accuracies for bronchiectasis segmentation using Mask R-CNN and van der Veer et al. demonstrated that automated quantification of mucus plugging on CT correlates with increased mortality in COPD [30], [31]. Extending these efforts toward clinical outcome prediction, Chassagnon et al. applied a deep learning model to quantify multiple structural abnormalities on CT in adults with CF, showing that bronchial wall thickening and mucus plugging were major predictors of lung function and exhibited marked reversibility following CFTR modulator therapy [32].

Recent advancements in automated lung segmentation on lung half and lung lobe level have strengthened efforts towards quantification of lung changes on MRI [33], [34]. Deep learning-based analysis of morphological lung changes such as bronchiectasis/wall thickening and mucus plugging has predominantly relied on CT [18]. Recent studies have begun to extend these analyses to MRI: either indirectly by converting MRI scans into synthetic CT images while maintaining key airway structures, or directly via UTE-MRI segmentation networks such as RiSeNet [35], [36]. Together, these studies highlight complementary strategies, that broaden the scope of radiation-free, deep learning–based lung analysis.

Despite challenges such as lower spatial resolution that can complicate post-processing, MRI’s superior soft-tissue contrast may enhance the visibility of airway wall changes and mucus plugging compared with CT, potentially facilitating automated assessment [28], [37], [8]. Building on these advances, this work presents a deep learning-based approach to classify bronchiectasis/wall thickening and mucus plugging in patients with cystic fibrosis using T2-weighted MRI scans.

## 2 Material and methods

### 2.1 Study cohort

This study is part of a prospective observational multicenter study (clinicaltrials.gov identifiers NCT00760071, NCT02270476), which was authorized by the institutional ethics committee and conducted in compliance with the Declaration of Helsinki and relevant scientific guidelines. Informed consent was obtained from all patients or their parents or legal guardians. The diagnosis of CF was confirmed by the presence of elevated sweat chloride (Cl-) concentrations (≥60 mmol/L) and CFTR mutation analysis. In patients with exocrine pancreatic sufficiency who have obtained inconclusive sweat test results (sweat Cl- concentrations of 30–59 mmol/L), the diagnosis was further supported by an assessment of CFTR function in rectal biopsies, as previously reported [38], [39]. Overall, 627 examinations from 164 patients were included in this study. Some patients were included in our previous reports on morpho-functional MRI [7], [8], [10].

### 2.2 Magnetic resonance imaging

Standardized chest MRIs were performed at an age of three months, and then continued annually as part of our monitoring programme using three 1.5 T scanner models from the same vendor (Magnetom Symphony, Magnetom Avanto and Magnetom Aera, Siemens Healthineers, Erlangen, Germany). The protocol for scanning remained essentially consistent throughout the study period as previously described [11], [7], [40], [9], [10]. A T2-weighted turbo spin echo sequence with rotating phase encoding (BLADE, Siemens Healthineers, Erlangen, Germany) was acquired in coronal and axial projections. In addition, T1-weighted sequences were obtained both prior and following the administration of intravenous contrast agent. Sedation was routinely administered to children aged five years or younger, either orally or rectally, using chloral hydrate (100 mg/kg body weight, maximum dose of 2 g) [8].

### 2.3 Chest MRI scoring system

**Fig. 1:**
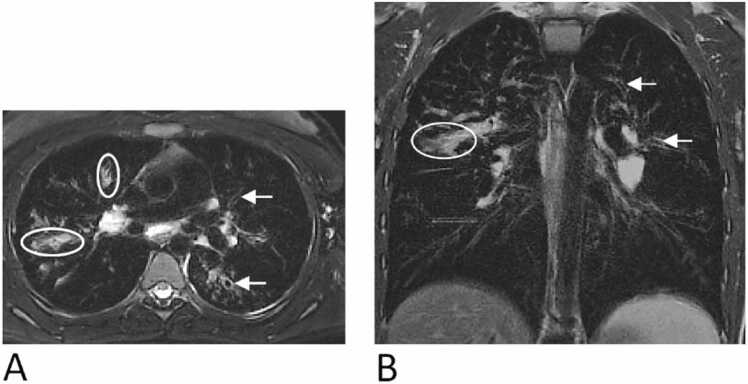
Axial (A) and coronal (B) slice of a patient with cystic fibrosis demonstrating bronchiectasis/wall thickening (white arrow) and mucus plugging (oval). Overall bronchiectasis/wall thickening score: 8; overall mucus plugging score: 6.

The present study was evaluated by an observer (MOW) specializing in chest MRI, with professional experience of over 15 years. The observer also evaluated all previous studies and assessed all MRI examinations using the Eichinger score based on the full MRI protocol [11], [8], [9], [10]. The chest MRI scoring system employs a numerical disease severity score to each lung lobe (e.g., 0 = absence, 1 = <50 % of a lobe affected, and 2 = ≥50 % of a lobe affected) for each of the morphological score items bronchiectasis/wall thickening, mucus plugging, sacculation/abscess, consolidation, special finding/pleural lesion and perfusion abnormalities [11]. The overall score for each item is defined as the sum of each lung lobe’s score and the lingula within a single examination, ranging from 0 to 12, representing the combined disease severity across all lobes. For the purpose of this study, we focused on the bronchiectasis/wall thickening and mucus plugging scores (Fig. 1). Due to the limited spatial resolution of the MRI, and as proposed by Eichinger et al. bronchiectasis and wall thickening were scored as a single parameter [11].

### 2.4 Dataset

The study comprised a total of 627 chest MRI examinations from 164 patients, with each patient contributing at least one examination (Table 1, Figure S1). Some patients underwent multiple scans over time, with a maximum of 16 examinations and a median of 4 examinations performed for a single patient. The study cohort ranged in age from 0.1 to 53 years with a median of 8.39 years (mean 7.03 ± 6.15 years), capturing both pediatric and adult CF populations. Overall, 41 MRI were collected from patients who were over 18 years old at the time of imaging. Of the 164 patients, 85 were male and 79 were female. Examinations were excluded if relevant imaging data were unavailable in the form of the BLADE sequence in either coronal or axial planes or the MRI score was missing (Figure S1). Imaging data from the T2-sequence were selected since this sequence is recommended for the detection of bronchiectasis/wall thickening and mucus plugging and was used for scoring [41], [11]. The dataset was divided into three sets: a training set, a validation set, and a test set, with each set comprising 60 %, 20 %, and 20 % of the total data, respectively. Thus, 131 patients (486 MRI) were allocated to the training and validation sets, with 99 patients (376 MRI) in training set and 32 patients (110 MRI) in validation. The remaining 33 patients (141 MRI) formed the test set. The test set was defined at the beginning of the study and held out for final evaluation.

**Table 1:** Overview of patient characteristics split by training/validation, internal test set and external test set. Values are provided as median [range] (SD). n corresponds to the number of observations per measurement.

	**Training / validation set**	**Internal test set**	**External test set**
**Patients**, n	131	33	10
**Cases**, n	486	141	10
**Age (y)** median [range] (std)	3.7 [0.1–53.0] ± 6.3	5.5 [1.1–34.6] ± 5.5	31.0 [14.6–49.3] ± 9.9
**Sex (m / f)**	66 / 65	19 / 14	6/4
**Height (cm)** median [range] (SD)	126.7 [57.8–194.0] ± 31.4	132.6 [52.7–182.9] ± 31.4	170.5 [156.5–187.0] ± 10.4
**Weight (kg]** median [range] (SD)	23.9 [4.0–83.0] ± 18.4	28.1 [8.7–72.0] ± 17.2	68.3 [52.6–97.5] ± 13.4
***CFTR*****genotype,** n (%) *F508del/F508del* *F508del/other* *Other/other*	129 (98) 57 (43) 56 (43) 16 (12)	33 (100) 16 (48) 13 (40) 4 (12)	10(100) 6(60) 3 (30) 1(10)
**Pancreatic insufficiency**, n (%)	107 (82)	27 (82)	9 (90)
**Spirometry,** n (%)	378 (78)	115 (82)	10 (100)
*Percent predicted FEV1* median [range] (std)	87.5 [38.8–125.8] ± 15.5	90.7 [26.1–122.4] ± 19.9	100 [62.4–110.8] ± 17.6
**Global MRI score** median [range] (SD)	12 [0−50] ± 7.8	11 [2−42] ±8.4	16.5 [6−32] ± 8.3
**MRI morphological score** median [range] (SD)	8 [0−40] ± 5.6	8 [1−31] ± 5.8	11.5 [6−23] ± 5.4
**Overall bronchiectasis/wall thickening score** median [range] (SD)	6 [0−12] ± 2.0	6 [1−12] ± 1.9	8.5 [5−11] ± 1.9
**Overall mucus plugging score** median [range] (SD)	2 [0−11] ± 2.3	1 [0−12] ± 2.3	6 [2−6] ± 1.6

In addition, an external test set consisting of BLADE MRI scans of ten patients was collected from a second centre (Table 1, external test set). The external patients were significantly older, with a median age of 31 years, and had overall advanced disease severity (median global MRI score: 16.5).

### 2.5 Distribution of scores in dataset

Given that the study cohort encompasses patients across a wide range of ages and disease severities, it is to be expected that the distribution of morphological scores for bronchiectasis/wall thickening and mucus plugging will naturally vary. The majority of mucus plugging scores fell into categories 0 or 1, while bronchiectasis/wall thickening was predominantly assigned a score of 1 (Table 2). A similar pattern emerged at the lobe level: most lobes exhibited either lower (0) or moderate (1) scores for both mucus plugging and bronchiectasis/wall thickening, with relatively fewer instances of severe involvement (2). For bronchiectasis/wall thickening, score 2 was the least frequent category in most lobes, except for the right upper lobe, where score 0 was the least common category.

**Table 2:** Distribution of scores of bronchiectasis/wall thickening and mucus plugging in all lung lobes and the lingula.

	Score	Left upper	Lingula	Left lower	Right upper	Right middle	Right lower	Overall
Bronchiectasis/ wall thickening	0	75	205	74	42	89	78	563
	1	496	374	515	465	464	511	2825
	2	56	48	38	120	74	38	374
Mucus plugging	0	412	433	377	291	363	375	2251
	1	187	175	240	260	235	241	1338
	2	28	19	10	76	29	11	173

### 2.6 Automated lung half segmentation and lobe approximation

All examinations underwent the following processing workflow: Each MRI volume first underwent bias-field correction with the N4ITK algorithm to reduce scanner-related inhomogeneities, and was then intensity-normalized using z-score normalization [42], [43].

Next, we used our previously trained nnU-Net to obtain coronal lung half segmentations, which were visually inspected and corrected if necessary [34]. The coronal segmentation masks were then resampled according to the information in the header of the corresponding axial MRI, thereby ensuring precise anatomical alignment. Because the coronal and axial scans were acquired in the same examination session and were inherently aligned, this resampling procedure accurately maintained anatomical fidelity in the axial plane, eliminating the need for additional registration. Following conversion into the axial plane, the morphological operation closing was applied to reduce edge artefacts introduced by slice thickness. The effect of this step was visually inspected across the dataset, confirming that segmentations remained anatomically consistent and that edge artefacts were minimized. Next, we applied an atlas-based approximation method, which we have previously used in a different project [44]. It is similar to that described by Tutison et al., utilizing the adapted 3D MRI lung model from BodyParts3D to estimate the placement of lung lobe fissures [45], [46]. The method transfers the lobe border information from a 3D lung model to individual patient lung anatomy combining rigid and elastic registration. The masks were then post-processed to close any holes that occurred during the transfer. For validation purposes, an experienced radiologist reviewed a subset of segmented lung lobes [47]. The final output of the segmentation pipeline were coronal and axial lung lobe approximations which were then used to crop the MRI.

### 2.7 Convolutional neural network architecture

**Fig. 2:**
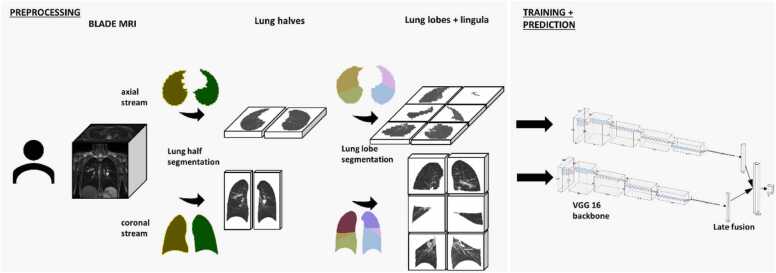
Processing pipeline of axial and coronal T2-weighted turbo spin echo sequence with rotating phase encoding MRI. Initially all MRI were processed with a lung half segmentation algorithm. Afterwards the lung lobes were approximated and cropped. Lastly, the lung lobes were fed into the corresponding stream of a dual-view variant of the VGG16 with custom classifier.

Our approach is based on an adapted dual-view variant of the VGG16 network, pre-trained on ImageNet, to classify lung changes at the lobe level [48]. We employed two parallel VGG16 streams, one for coronal slices and one for axial slices, enabling late fusion of complementary anatomical information similar to the method described by [49] (Fig. 2). Each stream processed a batch size of 1 (examination), with input dimension of [S x 3 × 256 x 256], where S represents the number of 2D slices in the MRI and 3 indicates the color channels introduced to match VGG16’s input requirements. Initially, each 2D slice passes through the standard VGG16 convolutional blocks, producing feature maps of size [S x 256 × 7×7]. Afterwards an adaptive average pooling layer (1 ×1) is applied to reduce the feature maps to [S x 256] [49]. To aggregate information across all slices in an examination, we perform a max-pooling step over the slice dimension, yielding a single 256-dimensional descriptor per patient and view. This step was necessary because Eichinger score labels are provided only at the lobe level rather than for individual slices. Finally, the embeddings from the coronal and axial streams undergo a late fusion step, where we concatenate the 256-dimensional descriptors from each view before feeding them into a linear classification layer predicting one of the three classes [50]. To address class imbalance, we introduced weighted loss functions, ensuring that majority classes receive higher penalty during training.

Data loading was managed by a custom PyTorch Dataset class. Coronal and axial images were stored as NumPy arrays, each transformed into a 3-channel format via channel repetition for VGG16. Intensity values were rescaled to [0, 1] enabling a consistent pipeline for reading, transforming, and batching paired images (Fig. 2). Online data augmentation was employed to increase dataset variability by dynamically applying random rotations (−180° to +180°) and horizontal or vertical flips (probability 0.5) during each training epoch.

### 2.8 Training process

To identify the most effective backbone architecture, we conducted an initial screening of several pre-trained convolutional neural networks, including AlexNet, ResNet18, ResNeXt50_32x4d, VGG16, EfficientNet-B3, and DenseNet121. Each model was evaluated based on its performance in predicting bronchiectasis/wall thickening (Tables S1.1-S1.6) and mucus plugging (Tables S2.1-S2.6) on a validation subset. Once VGG16 emerged as the top performer, we then focused on optimizing its hyperparameters. Ultimately, a step scheduler with a gamma of 0.4, an initial learning rate (LR) of 1e-5, and a step size of 15 yielded the highest accuracy and most consistent convergence. We used SGD as it converted faster than Adam. Consequently, VGG16 with a Step LR schedule and SGD at the specified learning rate settings was adopted as the final configuration for all subsequent experiments.

All configurations were trained for 125 epochs on two Tesla V100S PCIe 32 GB GPUs, with an average runtime of 22 min per epoch. Early stopping was triggered after 25 consecutive epochs without a decrease in validation loss.

### 2.9 Experiments

We evaluated our classification framework for bronchiectasis/wall thickening and mucus plugging using two distinct approaches: the Universal-Lobe Classifier and the Lobe-Expert Classifiers. In the Universal-Lobe Classifier approach, all lung lobes were consolidated into a single dataset and subjected to a five-fold cross-validation scheme, with no differentiation among lobe types during training. By presenting examples from any lobe to a single model, we assessed its ability to generalize across varying anatomical contexts. To derive an overall bronchiectasis/wall thickening and mucus plugging score, we aggregated the individual lobe predictions from each examination.

In contrast, the Lobe-Expert Classifiers approach treated each lobe category (left upper, lingula, left lower, right upper, right middle, and right lower) separately, implementing a two-fold cross-validation scheme for each due to computational limitations. This allowed the model to learn lobe-specific patterns and facilitated an evaluation of consistency across different anatomical regions. As with the Universal-Lobe Classifier approach, per-lobe predictions were ultimately aggregated to estimate the whole-lung bronchiectasis/wall thickening and mucus plugging scores.

### 2.10 Data splitting

We partitioned the dataset at the patient level to ensure that all examinations from each patient remained together in a single set, thus preserving patient independence across training, validation and test. Each patient contributed multiple examinations with scores of 0, 1 or 2, and our goal was to balance these as evenly as possible in a 60 %/20 %/20 % ratio. To accomplish this, we implemented an iterative random-search algorithm that, on each iteration, shuffled the patient list and assigned the patients to one of the three sets. For every iteration, we computed a scalar cost – the sum of absolute deviations between the observed score counts and the ideal count. After all iterations, the assignment with the lowest cost was selected as our final patient-level split, yielding the most balanced distribution of scores across all three sets.

### 2.11 Grad-CAM visualization

To interpret the model’s predictions, we applied Gradient-weighted Class Activation Mapping (Grad-CAM) to visualize regions contributing most to the predicted class [51]. Grad-CAM was computed from the last convolutional layer of each VGG16 branch (coronal and transversal), using the gradient of the predicted class score to weight the feature maps. The resulting heatmaps were normalized to [0,1], upsampled to the original slice resolution, and thresholded to highlight highly relevant regions. Slice-wise Grad-CAM maps were resampled into 3D volumes of the entire lung for direct comparison with the original MRI. For validation, both pathologies were highlighted in axial MRI and visualized together with the Grad-CAM overlay for interpretation.

### 2.12 External validation

Model generalizability was assessed on the independent external test set comprising 10 patients from a different center (Table 1). The Universal-Lobe Classifier was applied directly to the external data as inference using the same preprocessing and scoring pipelines. Predictions were compared to reference scores using the same set of metrics as for internal validation. No model re-training or fine-tuning was performed on this dataset.

### 2.13 Statistical analyses

For per-lobe predictions (scores 0, 1, and 2), model performance was evaluated using sensitivity, specificity, accuracy, class-wise calibration error (CWCE), and class-wise AUROC in a one-vs-rest manner [52]. Agreement beyond chance was quantified with quadratic weighted Cohen’s κ and Gwet’s AC2, both suitable for ordinal data [53], [54]. Discrimination was further summarized using macro AUROC across all classes, and calibration using macro CWCE.

For the overall lung score (0–12), agreement between predicted and reference scores was assessed with the intraclass correlation coefficient (ICC(2,1)) and Pearson’s correlation coefficient. In addition, Bland–Altman analysis was used to compute mean differences and 95 % limits of agreement (LoA), allowing direct comparison with established inter- and intra-rater variability from manual scoring. This mirrors the methodology employed in the original Eichinger score validation [11].

All analyses were conducted in Python (version 3.9) using SciPy (version 1.11.4) [55]. Results are reported as mean ± standard deviation (SD) across cross-validation folds, with 95 % confidence intervals provided where applicable. Statistical significance was set at p < 0.001. Results are presented following five-fold cross-validation for the Universal-Lobe Classifier and two-fold cross-validation for the Lobe-Expert Classifiers.

## 3 Results

### 3.1 Encouraging Universal-Lobe Classifier results for bronchiectasis/wall thickening

For bronchiectasis wall thickening, the Universal-Lobe-Classifier achieved moderate agreement across folds, with quadratic weighted kappa and Gwet’s AC2 both around 0.53. Discrimination performance was strong, with a macro AUROC of 0.87, while calibration error remained low (macro CWCE ≈ 0.032; Table 4).

**Table 3:** Per-class and overall prediction performance of the Universal-Lobe Classifier for bronchiectasis/wall thickening. Metrics are reported as mean [95 % CI]; Asterisks indicate statistical significance at p < 0.001.

	Score 0	Score 1	Score 2	Overall
Accuracy	0.869 [0.858, 0.878]	0.807 [0.795, 0.819]	0.939 [0.931, 0.945]	-
Sensitivity	0.240 [0.206, 0.277]	0.931 [0.922, 0.940]	0.653 [0.611, 0.693]	-
Specificity	0.964 [0.957, 0.969]	0.438 [0.408, 0.468]	0.978 [0.972, 0.982]	-
AUROC	0.828 [0.808, 0.848] *	0.806 [0.739, 0.873] *	0.972 [0.960, 0.984] *	Macro: 0.870 ± 0.007
CWCE	0.0273 [0.022, 0.0379]	0.0303 [0.0249, 0.0438]	0.0171 [0.0121, 0.0238]	Macro: 0.0317 ± 0.0085
Quadratic weighted kappa	-	-	-	0.533 ± 0.022
Gwet’s AC2	-	-	-	0.532 ± 0.022

**Table 4:** Results for overall lobe scoring (0−12) for bronchiectasis/wall thickening. Asterisks indicate statistical significance at p < 0.001.

	Value	95 % CI
ICC(2,1)	0.690	0.650–0.724
Pearson r	0.739*	0.704–0.768

On the per-class level, the model showed high accuracy for scores 0 and 2, but lower accuracy for score 1 (Table 4). Sensitivity was highest for score 1 (0.93) but substantially lower for scores 0 and 2, reflecting some class-specific imbalance. Specificity was high for scores 0 and 2 but lower for score 1. AUROC values ranged from 0.81 to 0.97 across classes, confirming good discrimination between true and predicted labels.

The normalized confusion matrix for bronchiectasis/wall thickening (Fig. 5) shows that the classifier most reliably identified lobes with changes affecting less than 50 % of the lobe (score 1). Whereas discrimination cases with no bronchiectasis/wall thickening (score 0) and those with more extensive involvement affecting equal or more thatn 50 % of the lobe (score 2) proved more challenging, with frequent misclassification of severe cases as moderate.

When aggregating per-lobe predictions into an overall score ranging from 0 to 12, the model demonstrated good consistency with expert ratings. The ICC(2,1) was 0.69 and the Pearson correlation was 0.74, statistically significant with narrow confidence intervals (Table 4). Bland–Altman analysis showed a small positive mean difference of 0.28 between predicted and reference scores, with 95 % limits of agreement ranging from −2.66 to 3.21 (Fig. 6).

**Fig. 3:**
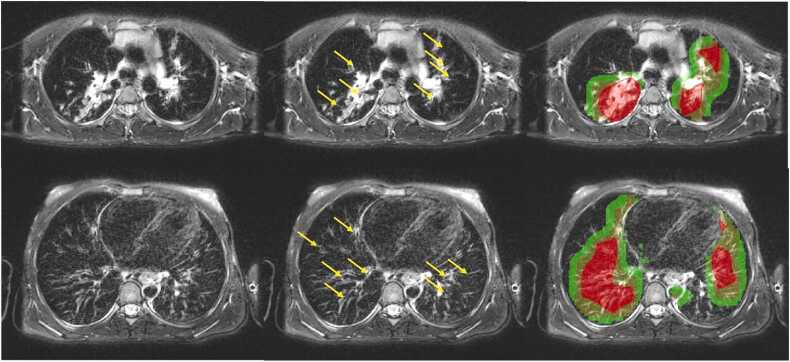
Grad-CAM visualization for bronchiectasis/wall thickening. The figure shows (from left to right): the original MRI, the MRI with highlights of bronchiectasis/wall thickening (yellow arrows) and an overlay displaying Grad-CAM. Grad-CAM relevance is color-coded, with red indicating highest relevance, yellow moderate relevance, and green lower relevance.

In Fig. 3 two representative axial MRI slices from the same patient at different anatomical levels are shown. The overlays demonstrate that the Grad-CAM activations are predominantly located within the lung regions and often correspond to areas of bronchiectasis/wall thickening, indicating that the model focuses on clinically relevant structures.

### 3.2 Universal-Lobe Classifier performs well for mucus plugging

The Universal-Lobe Classifier achieved high accuracy across all mucus plugging severity classes, with the best discrimination for score 2 (AUROC 0.98) and slightly lower values for score 1 (AUROC 0.84). Sensitivity was highest for the absence of plugging (score 0), while specificity peaked for severe plugging (score 2). Calibration was best for severe plugging, whereas mild and moderate scores showed slightly higher miscalibration (Table 5).

**Table 5:** Per-class and overall prediction performance of the Universal-Lobe Classifier for mucus plugging. Metrics are reported as mean [95 % CI]; Asterisks indicate statistical significance at p < 0.001.

	Score 0	Score 1	Score 2	Overall
Accuracy	0.830 [0.819, 0.840]	0.811 [0.800, 0.822]	0.981 [0.977, 0.985]	-
Sensitivity	0.871 [0.859, 0.882]	0.680 [0.655, 0.705]	0.691 [0.617, 0.756]	-
Specificity	0.741 [0.718, 0.763]	0.862 [0.850, 0.873]	0.992 [0.989, 0.994]	-
AUROC	0.874 [0.830, 0.919] *	0.836 [0.815, 0.856] *	0.982 [0.971, 0.992] *	Macro: 0.898 ± 0.004
CWCE	0.0925 [0.0834, 0.1031]	0.0875 [0.0774, 0.0982]	0.0062 [0.0037, 0.0104]	Macro: 0.0645 ± 0.0120
Quadratic weighted kappa	-	-	-	0.679 ± 0.009
Gwet’s AC2	-	-	-	0.679 ± 0.009

Across folds, the Universal-Lobe-Classifier demonstrated substantial agreement, with both quadratic weighted kappa and Gwet’s AC2 averaging 0.68. Discrimination performance was strong, reflected by a macro AUROC of 0.90, while calibration error remained low with a macro CWCE of 0.065 (Table 5).

The confusion matrix for mucus plugging (Fig. 5) illustrates that the model most reliably identifies lobes without mucus plugging (score 0), while lobes with changes affecting less than 50 % of the lobe (score 1) were more frequently confused with neighboring categories.

When predictions were aggregated into an overall mucus plugging score (0–12), the model demonstrated excellent agreement with the reference standard, with both ICC(2,1) and Pearson correlation reaching 0.84 (p < 0.001) (Table 6). Bland–Altman analysis (Fig. 6) confirmed minimal bias between predicted and true scores, indicating good reliability of the overall score and narrow limits of agreement from −2.66–2.72.

**Table 6:** Results for overall lobe scoring (0−12) for mucus plugging. Asterisks indicate statistical significance at p < 0.001.

	Value	95 % CI
ICC(2,1)	0.843	0.814–0.867
Pearson r	0.843*	0.814–0.867

**Fig. 4:**
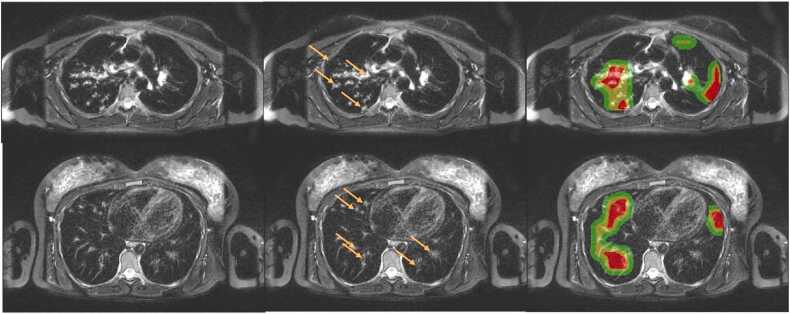
Grad-CAM visualization for mucus plugging. The figure shows (from left to right): the original MRI, the MRI with highlights of mucus plugging (orange arrows) and Grad-CAM an overlay displaying Grad-CAM. Grad-CAM relevance is color-coded, with red indicating highest relevance, yellow moderate relevance, and green lower relevance.

Fig. 4 illustrates Grad-CAM visualizations for a patient with mucus plugging. The Grad-CAM activations highlight areas that contributed most to the model’s predictions and show a correspondence with the highlighted pathological regions. This alignment suggests that the network focuses on clinically meaningful features and supports the interpretability and reliability of the model’s decision-making process.

**Fig. 5:**
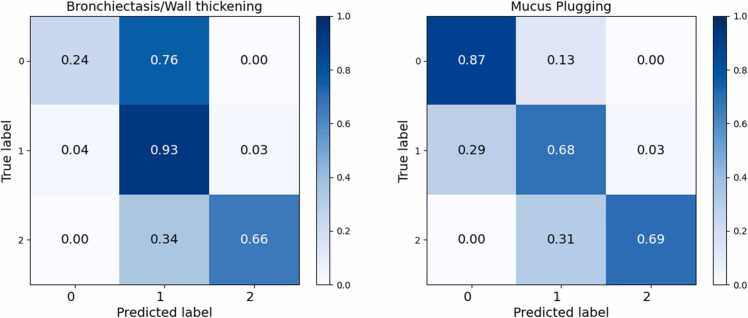
Normalized confusion matrix after five-fold cross validation for lung lobe-based prediction of bronchiectasis/wall thickening and mucus plugging.

**Fig. 6:**
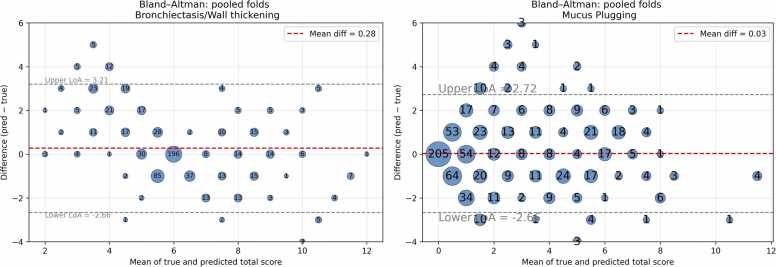
Bland-Altmann plots for bronchiectasis/wall thickening and mucus plugging.

### 3.3 Lobe-Based Classifiers show inferior performance

Lobe-Expert Classifiers for bronchiectasis/wall thickening and mucus plugging showed heterogeneous and generally weaker performance compared to the Universal-Lobe approach. Accuracy and agreement varied considerably across lobes, with some regions approaching random performance while others achieved moderate correlations with reference scores. For mucus plugging, predictions tended to underestimate severity, with a systematic negative bias and broad limits of agreement. These findings indicate that training independent models per lobe does not improve performance and instead introduces instability, likely due to class imbalance and variable disease patterns across lobes. Detailed per-lobe results are provided in the Supplement (bronchiectasis/wall thickening: Tables S3.1–S3.7, Figures S2.1–S2.6; mucus plugging: Tables S4.1–S4.7, Figures S3.1–S3.6).

### 3.4 External validation suggests promising generalization

In the external validation cohort (n = 10), the Universal-Lobe Classifier maintained moderate agreement and acceptable discrimination for both pathologies despite the limited sample size. For bronchiectasis/wall thickening, agreement with reference scores was fair (quadratic weighted κ = 0.26; Gwet’s AC2 = 0.25), and discrimination performance was moderate (macro AUROC = 0.77) with a higher calibration error (macro CWCE = 0.14) compared to internal results (Table 7).

**Table 7:** Prediction performance of the Universal-Lobe Classifier for bronchiectasis/wall thickening on the external dataset. Metrics are averaged across folds and reported as mean [95 % CI].

	Score 0	Score 1	Score 2	Overall
Accuracy	0.983 [0.911, 0.997]	0.617 [0.490, 0.729]	0.633 [0.507, 0.744]	-
Sensitivity	0.000 [0.000, 0.793]	0.774 [0.602, 0.886]	0.464 [0.295, 0.642]	-
Specificity	1.000 [0.939, 1.000]	0.448 [0.284, 0.625]	0.781 [0.612, 0.890]	-
AUROC	nan [nan, nan] (p = nan)	0.700 [0.410, 0.989] (p = 0.176)	0.796 [0.559, 1.000] (p = 0.0143)	Macro: 0.770
CWCE	0.0637 [0.0423, 0.1087]	0.1655 [0.1775, 0.3545]	0.1973 [0.1583, 0.3398]	Macro: 0.1421
Quadratic weighted kappa	-	-	-	0.258
Gwet’s AC2	-	-	-	0.248

**Fig. 7:**
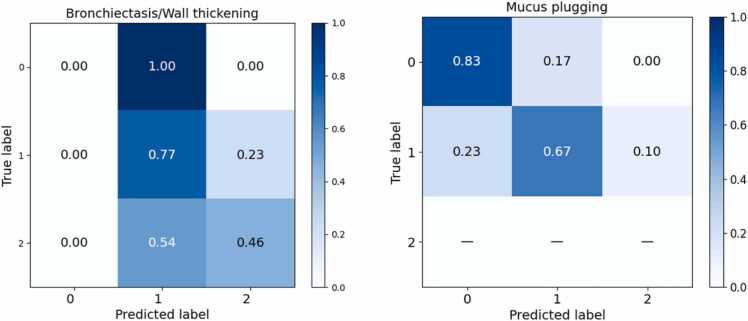
Normalized confusion matrix for lung lobe-based prediction of bronchiectasis/wall thickening and mucus plugging on the external dataset.

The confusion matrix for bronchiectasis/wall thickening shows that nearly all lobes with score 0 were misclassified as score 1, while lobes with score 1 were generally well recognized (77 %), but some were predicted as score 2 (Fig. 7). Lobes with score 2 were often underestimated as score 1 (54 %), with only 46 % correctly classified.

Correlation for overall scores remained reasonable (ICC(2,1) = 0.54; Pearson’s r = 0.54), though wide confidence intervals reflect the small cohort size. AUROC values for score 0 could not be computed due to the absence of positive cases in that category (Table 8).

**Table 8:** Results for overall lobe scoring (0−12) for bronchiectasis/wall thickening on the external dataset.

	Value	95 % CI
ICC(2,1)	0.543	0.0–0.853
Pearson r	0.549	0.0–0.880 (p = 0.1)

For mucus plugging, performance was comparable, showing moderate agreement (κ = 0.43; AC2 = 0.43) and good discrimination (macro AUROC = 0.83) (Table 9). Calibration remained consistent (macro CWCE = 0.20), and the overall score correlation was strong (ICC(2,1) = 0.68; Pearson’s r = 0.76) (Table 10). Missing AUROC values for score 2 reflect class imbalance in the external dataset. Overall, while calibration and agreement metrics were lower than internal cross-validation, the classifier preserved robust discrimination and score-level correlation, supporting its generalization to unseen data.

**Table 9:** Prediction performance of the Universal-Lobe Classifier for mucus plugging on the external dataset. Metrics are averaged across folds and reported as mean [95 % CI].

	Score 0	Score 1	Score 2	Overall
Accuracy	0.783 [0.664, 0.869]	0.700 [0.575, 0.801]	0.917 [0.819, 0.964]	-
Sensitivity	0.833 [0.552, 0.953]	0.667 [0.525, 0.783]	nan [nan, nan]	-
Specificity	0.771 [0.635, 0.867]	0.833 [0.552, 0.953]	0.917 [0.819, 0.964]	-
AUROC	0.875 [0.722, 1.000] *	0.793 [0.011, 1.000] (p = 0.462)	nan [nan, nan] (p = nan)	Macro: 0.834
CWCE	0.1559 [0.1118, 0.2632]	0.2912 [0.2615, 0.4082]	0.1634 [0.1140, 0.2197]	Macro: 0.203
Quadratic weighted kappa	-	-	-	0.430
Gwet’s AC2	-	-	-	0.428

**Table 10:** Results for overall lobe scoring (0−12) for bronchiectasis/wall thickening on the external dataset.

	Value	95 % CI
ICC(2,1)	0.682	0.095–0.865
Pearson r	0.762	0.203–0.985 (p = 0.0104)

In the mucus plugging confusion matrix, lobes with score 0 were classified correctly in the majority of cases (83 %), while score 1 lobes were predicted correctly in 67 % of cases, with some misclassified as score 0. Score 2 cases were absent in this external dataset, leaving this row empty (Fig. 7).

## 4 Discussion

Our aim was to develop and validate a deep learning-based approach for the automated classification of bronchiectasis/wall thickening and mucus plugging on T2-weighted MRI. We implemented two strategies for each pathology: a Universal-Lobe Classifier trained on all lobes together, and Lobe-Expert Classifiers trained separately for each lobe. Predictions were evaluated on both the lobe (scores 0–2) and lung (scores 0–12) level. The models achieved higher agreement and discrimination for mucus plugging than for bronchiectasis/wall thickening and the Universal-Lobe Classifier outperformed the Lobe-Expert Classifiers across both pathologies. Quadratic weighted κ indicated moderate to substantial agreement on the internal dataset, consistent with reported inter-reader variability. Grad-CAM visualizations confirmed that predictions were based on anatomically and clinically plausible regions. Evaluation on an external test set showed consistent generalization, with lower κ values likely reflecting domain differences and the limited sample size rather than model instability. To interpret these findings in context, we first discuss the rationale for focusing on these two scores and their clinical relevance.

Our analysis focused on bronchiectasis/wall thickening and mucus plugging due to their prevalence and more balanced distribution in the available MRI data, which enabled a robust AI-based assessment. These scores also represent the most common morphological lung changes in pediatric and clinically stable patients with cystic fibrosis [40], [10]. Other components of the Eichinger score, such as consolidations, abscesses/sacculations, and special findings, showed highly imbalanced distributions in our dataset, effectively limiting their suitability for supervised learning with the present sample size. Perfusion scoring was not included, as it requires multi-sequence dynamic imaging. Future work should extend this approach to additional scores and perfusion analysis once larger and more diverse datasets become accessible.

Our findings indicate that automated lobe-level scoring of CF-related lung changes on MRI is feasible and may serve as a supportive tool in clinical workflows. By providing consistent and reproducible predictions based on standardized and clinically established image features, the model could assist radiologists in routine reporting. The approach is intended to complement, rather than replace, expert assessment by offering an initial, objective estimation that can help focus manual review to relevant areas. In the long term, such tools may contribute to more standardized evaluations and facilitate quantitative comparisons across longitudinal studies, multi-center research and routine clinical care.

To better understand the observed performance differences across pathologies, we analyzed factors affecting classification outcomes. The observed performance differences between mucus plugging and bronchiectasis/wall thickening can be explained by several factors. First, class imbalance was more pronounced for bronchiectasis/wall thickening, with most lobes receiving intermediate scores, limiting the model’s ability to learn discriminative features for rare mild (score 0) or severe cases (score 2) [56]. In contrast, mucus plugging exhibited a more balanced distribution across severity levels, likely contributing to its superior classification performance. These imbalances reflect the underlying disease distribution in CF, where mild-to-moderate structural changes are common, while severe manifestations have become less frequent due to advances in therapy. Second, the coarse 0/1/2 scoring scale restricts granularity and may obscure subtle variations in disease extent. For instance, assigning the same score to both minimal and extensive findings, or treating a small and a large lobe equally despite their differing physiological impact. Third, MRI’s limited spatial resolution prevents reliable separation of bronchiectasis and wall thickening, which are scored jointly in the Eichinger system, introducing additional label ambiguity. Together, these factors likely contributed to the comparatively lower agreement for bronchiectasis/wall thickening, as reflected in the per-lobe metrics summarized in Table 3, Table 4. Future work could explore more granular scoring systems, patch-based classification, or data balancing techniques such as augmentation or synthetic data generation to mitigate these limitations and improve recognition of rare pathological states [57], [58]. Crisosto et al. described an approach that artificially generates consolidations on MRI to balance the dataset for U-Net–based lung parenchyma segmentation, resulting in improved performance with the inclusion of synthetic data [59]. This method could be a promising direction for future research in addressing class imbalances in CF imaging.

Compared with prior CT-based studies achieving high accuracy in structural lung abnormality detection, our MRI-based approach attains comparable reproducibility, particularly for mucus plugging, despite the lower spatial resolution and coarser label granularity inherent to MRI. In the original Eichinger score publication, inter-reader limits of agreement reached approximately ±2–3 points for both bronchiectasis/wall thickening and mucus plugging, each assessed on a 0–12 scale, a variability later confirmed by subsequent studies [11], [40]. Our automated predictions fall within this range, with mean differences close to zero and limits of agreement between roughly −2.7 and + 3.2 points, indicating that the model’s reproducibility is on a similar level to expert readers. The mean bias for mucus plugging (≈ 0.03) was even smaller than those reported for human observers, suggesting minimal systematic deviation. This is particularly encouraging given that the model was trained on human-generated labels and thus inherits their variability. Importantly, once trained, the system produces fully consistent outputs, eliminating intra-reader fluctuations due to fatigue, context, or time. In contrast to recent MRI-based works focusing on voxel-level segmentation with paired MRI-CT data, our study shows that clinically meaningful, reproducible classification can also be achieved from routine, weakly labeled MRI data, highlighting the feasibility of label-efficient, reproducible CF lung assessment [35], [36]. In addition to reproducing human-level variability, our method demonstrates strong interpretability and promising generalization, as discussed below.

To enhance interpretability, Grad-CAM visualizations were generated for a subset of cases. Additionally, manual highlighting of the pathologies was conducted by a radiologist to support visual interpretation. Joint visualization showed that the model predominantly focused on anatomically and clinically plausible regions corresponding to mucus plugs or bronchial wall abnormalities. These overlays support that predictions are driven by meaningful image features rather than spurious correlations. Generalization was further assessed on an external dataset comprised of ten patients from a different centre. As expected, performance metrics declined due to the small sample size and domain differences, yet the trends in agreement and discrimination remained directionally consistent with internal validation. These findings suggest that the proposed approach captures transferable imaging patterns and may serve as a foundation for multi-centre training and validation efforts to improve robustness across diverse scanners and acquisition protocols. While these findings are encouraging, certain architectural and methodological factors also influenced model behavior, which are outlined next.

The dual-view late fusion architecture was designed to integrate complementary information from coronal and axial slices, reflecting radiological practice and enabling robust extraction of global lobe-level features. However, because features are aggregated through slice-wise pooling with limited spatial supervision, some spatial imprecision may occur. This may explain occasional mismatches between Grad-CAM highlights and true lesion locations. The use of a batch size of one, required for full-volume processing, may further introduce training instability due to the absence of stable batch statistics. In parallel, the atlas-based lobe approximation can be sensitive to severe structural alterations such as collapse, hyperinflation, or scarring, leading to registration errors and misassigned regions that propagate through classification. Given the predominance of milder disease in our cohort, these inaccuracies likely had a limited overall effect, but they highlight the need for more robust segmentation methods, potentially deep learning-based approaches that adapt to diverse lung morphologies [33]. Finally, the reliance on weak lobe-level labels rather than voxel-level annotations constrains spatial precision and interpretability. Previous work has demonstrated the potential of detailed segmentation for assessing regional disease manifestations, but such annotations require extensive expert effort and remain impractical for large cohorts [28]. Integrating finer-grained annotations in future work could enhance localization and further align predictions with pathology.

Our study has several limitations. The single-centre, retrospective design may limit generalizability, underscoring the need for larger, multi-centre studies to capture broader variability in acquisition protocols and disease presentations. All MRIs were annotated by a single experienced radiologist, preventing a direct assessment of inter-reader variability. Incorporating multi-reader consensus labels in future work would allow more robust benchmarking of model performance against expert agreement. The exclusive focus on T2-weighted BLADE sequences and the coarse three-point Eichinger scale per lobe, restricted to the bronchiectasis/wall thickening and mucus plugging scores, narrows the scope of disease characterization but reflects current clinical routine and data availability. Another potential limitation is the presence of multiple examinations per patient, which may increase the risk of overfitting to individual patient characteristics. Despite these limitations, the approach offers a foundation for future research.

Looking ahead, in addition to using imaging data from different sequences, integrating results of clinical tests into the classification framework could provide additional context and improve prognostic accuracy. Developing lobe-specific or patient-tailored models that account for variations in age, disease severity and gender may further refine predictions but will require larger, more diverse datasets. Broader multicenter evaluation is essential to ensure robustness across imaging sites and scanner types. With the increasing adoption of highly effective CFTR modulators, the clinical phenotype of CF is shifting toward milder disease courses, which may also influence imaging strategies, potentially reducing the frequency of MRI follow-up or motivating the development of new, more sensitive MRI sequences tailored to subtle residual disease. Automated tools like the one proposed here could support such evolving protocols by enabling consistent, quantitative assessments even as imaging indications change. Future research should also explore voxel-wise annotations and hybrid approaches that combine lobe-level scoring with regional or lesion-specific analysis, potentially enhancing spatial precision and interpretability. As the methodology matures and achieves clinically comparable and validated performance, its translation into a medical product following appropriate standards and regulatory frameworks could enable deployment in routine care.

In summary, this study demonstrates the feasibility of a deep learning-based approach for the automated assessment of CF-related lung changes on MRI, focusing on bronchiectasis/wall thickening and mucus plugging. The results show that consistent, reproducible lobe-level scoring is achievable, laying the groundwork for more objective and efficient evaluation of structural lung abnormalities. With further validation and refinement, such methods could support standardized, reproducible assessments in clinical workflows and longitudinal studies, ultimately contributing to improved clinical management of CF.

## CRediT authorship contribution statement

**Mark O. Wielpütz:** Writing – review & editing, Writing – original draft, Supervision, Resources, Funding acquisition, Data curation. **Friedemann G. Ringwald:** Writing – review & editing, Writing – original draft, Visualization, Validation, Software, Resources, Methodology, Investigation, Formal analysis, Data curation, Conceptualization. **Urs Eisenmann:** Writing – review & editing, Writing – original draft, Supervision, Resources, Conceptualization. **Lena Wucherpfennig:** Writing – review & editing, Investigation, Funding acquisition, Data curation. **Knaup-Gregori Petra:** Writing – review & editing, Supervision, Resources, Funding acquisition. **Mirjam Stahl:** Writing – review & editing, Validation, Resources. **Olaf Sommerburg:** Writing – review & editing, Resources, Data curation. **Marcus A. Mall:** Writing – review & editing, Resources, Data curation. **Simon Y. Graeber:** Writing – review & editing, Resources, Data curation. **Anna Martynova:** Writing – review & editing, Methodology. **Niclas Hagen:** Writing – original draft, Supervision, Investigation. **Jacqueline Kürschner:** Writing – review & editing, Data curation. **Shengkai Zhao:** Writing – review & editing, Methodology.

## Declaration of generative AI and AI-assisted technologies in the writing process

During the preparation of this work the authors used DeepL Write and ChatGPT in order to improve readability. After using these tools, the authors reviewed and edited the content as needed and take full responsibility for the content of the published article.

## Declaration of Competing Interest

The authors declare the following financial interests/personal relationships which may be considered as potential competing interests: Lena Wucherpfennig reports financial support was provided by Vertex Pharmaceuticals. Mark O. Wielpuetz reports financial support was provided by Vertex Pharmaceuticals. Marcus A. Mall reports financial support was provided by Vertex Pharmaceuticals. Marcus A. Mall reports financial support was provided by German Research Foundation. Marcus A. Mall reports financial support was provided by German federal Ministry for Education and Research. Mark O. Wielpuetz reports a relationship with Boehringer Ingelheim GmbH that includes: consulting or advisory. Lena Wucherpfennig reports a relationship with Antaros Medical that includes: consulting or advisory. Marcus A. Mall reports a relationship with Boehringer Ingelheim GmbH that includes: board membership, consulting or advisory, and travel reimbursement. Marcus A. Mall reports a relationship with Enterprise Therapeutics Ltd that includes: board membership and consulting or advisory. Marcus A. Mall reports a relationship with Kither Biotech that includes: board membership and consulting or advisory. Marcus A. Mall reports a relationship with SpliSense that includes: consulting or advisory. Marcus A. Mall reports a relationship with Vertex Pharmaceuticals that includes: board membership, speaking and lecture fees, and travel reimbursement. Marcus A. Mall reports a relationship with Pari that includes: board membership. If there are other authors, they declare that they have no known competing financial interests or personal relationships that could have appeared to influence the work reported in this paper.

## References

[1] Bell S.C., Mall M.A., Gutierrez H., Macek M., Madge S., Davies J.C. (2020). The future of cystic fibrosis care: a global perspective. Lancet Respir Med.

[2] Mall M.A., Burgel P.-R., Castellani C., Davies J.C., Salathe M., Taylor-Cousar J.L. (2024). Cystic fibrosis. Nat Rev Dis Prim.

[3] Mall M.A., Hartl D. (2014). CFTR: cystic fibrosis and beyond. Eur Respir J.

[4] Graeber S.Y., Mall M.A. (2023). The future of cystic fibrosis treatment: from disease mechanisms to novel therapeutic approaches. Lancet.

[5] Mall M.A., Mayer-Hamblett N., Rowe S.M. (2020). Cystic fibrosis: emergence of highly effective targeted therapeutics and potential clinical implications. Am J Respir Crit Care Med.

[6] Shteinberg M., Haq I.J., Polineni D., Davies J.C. (2021). Cystic fibrosis. Lancet.

[7] Stahl M., Steinke E., Graeber S.Y., Joachim C., Seitz C., Kauczor H.-U. (2021). Magnetic resonance imaging detects progression of lung disease and impact of newborn screening in preschool children with cystic fibrosis. Am J Respir Crit Care Med.

[8] Wielpütz M.O., Puderbach M., Kopp-Schneider A., Stahl M., Fritzsching E., Sommerburg O. (2014). Magnetic resonance imaging detects changes in structure and perfusion, and response to therapy in early cystic fibrosis lung disease. Am J Respir Crit Care Med.

[9] Wielpütz M.O., Stackelberg O. von, Stahl M., Jobst B.J., Eichinger M., Puderbach M.U. (2018). Multicentre standardisation of chest MRI as radiation-free outcome measure of lung disease in young children with cystic fibrosis. J Cyst Fibros.

[10] Wielpütz M.O., Stahl M., Triphan S.M.F., Wucherpfennig L., Leutz-Schmidt P., Gestewitz S. (2024). Longitudinal magnetic resonance imaging of changes in lung morphology and perfusion in children with cystic fibrosis from infancy through adolescence. Ann Am Thorac Soc.

[11] Eichinger M., Optazaite D.-E., Kopp-Schneider A., Hintze C., Biederer J., Niemann A. (2012). Morphologic and functional scoring of cystic fibrosis lung disease using MRI. Eur J Radio.

[12] Graeber S.Y., Renz D.M., Stahl M., Pallenberg S.T., Sommerburg O., Naehrlich L. (2022). Effects of Elexacaftor/Tezacaftor/Ivacaftor therapy on lung clearance index and magnetic resonance imaging in patients with cystic fibrosis and one or two F508del alleles. Am J Respir Crit Care Med.

[13] Stahl M., Dohna M., Graeber S.Y., Sommerburg O., Renz D.M., Pallenberg S.T. (2024). Impact of elexacaftor/tezacaftor/ivacaftor therapy on lung clearance index and magnetic resonance imaging in children with cystic fibrosis and one or two F508del alleles. Eur Respir J.

[14] Stahl M., Roehmel J., Eichinger M., Doellinger F., Naehrlich L., Kopp M.V. (2023). Effects of Lumacaftor/Ivacaftor on cystic fibrosis disease progression in children 2 through 5 years of age homozygous for F508del-CFTR: a phase 2 Placebo-controlled clinical trial. Ann Am Thorac Soc.

[15] Wucherpfennig L., Triphan S.M.F., Wege S., Kauczor H.-U., Heussel C.P., Sommerburg O. (2023). Elexacaftor/Tezacaftor/Ivacaftor improves bronchial artery dilatation detected by magnetic resonance imaging in patients with cystic fibrosis. Ann Am Thorac Soc.

[16] Litjens G., Kooi T., Bejnordi B. Ehteshami, Setio A. Arindra Adiyoso, Ciompi F., Ghafoorian M. (2017). A survey on deep learning in medical image analysis. Med Image Anal.

[17] Ranschaert Erik R., Morozov Sergey, Algra Paul R. (2019).

[18] Astley J.R., Wild J.M., Tahir B.A. (2022). Deep learning in structural and functional lung image analysis. Br J Radio.

[19] San José Estépar R. (2022). Artificial intelligence in functional imaging of the lung. Br J Radio.

[20] Pakzad A., Jacob J. (2022). Radiology of bronchiectasis. Clin Chest Med.

[21] Tepper L.A., Utens E.M.W.J., Caudri D., Bos A.C., Gonzalez-Graniel K., Duivenvoorden H.J. (2013). Impact of bronchiectasis and trapped air on quality of life and exacerbations in cystic fibrosis. Eur Respir J.

[22] Turkovic L., Caudri D., Rosenow T., Hall G., Stick S. (2017). Cystic Fibrosis. ERS International Congress 2017 abstracts.

[23] Dhar T., Dey N., Borra S., Sherratt R.Simon (2023). Challenges of deep learning in medical image Analysis—Improving explainability and trust. IEEE Trans Technol Soc.

[24] Lassen B., van Rikxoort E.M., Schmidt M., Kerkstra S., van Ginneken B., Kuhnigk J.-M. (2013). Automatic segmentation of the pulmonary lobes from chest CT scans based on fissures, vessels, and bronchi. IEEE Trans Med Imaging.

[25] Peng Y., Zhong H., Xu Z., Tu H., Li X., Peng L. (2021). Pulmonary lobe segmentation in CT images based on lung anatomy knowledge. Math Probl Eng.

[26] Sun J., Liao X., Yan Y., Zhang X., Sun J., Tan W. (2022). Detection and staging of chronic obstructive pulmonary disease using a computed tomography-based weakly supervised deep learning approach. Eur Radio.

[27] Walsh S.L.F., Mackintosh J.A., Calandriello L., Silva M., Sverzellati N., Larici A.Rita (2022). Deep Learning-based outcome prediction in progressive fibrotic lung disease using High-Resolution computed tomography. Am J Respir Crit Care Med.

[28] Dournes G., Hall C.S., Willmering M.M., Brody A.S., Macey J., Bui S. (2022). Artificial intelligence in computed tomography for quantifying lung changes in the era of CFTR modulators. Eur Respir J.

[29] Aliboni L., Pennati F., Gelmini A., Colombo A., Ciuni A., Milanese G. (2022). Detection and classification of bronchiectasis through convolutional neural networks. J Thorac Imaging.

[30] van der Veer T., Andrinopoulou E.-R., Braunstahl G.-J., Charbonnier J.Paul, Kim V., Latisenko R. (2025). Association between automatic AI-based quantification of airway-occlusive mucus plugs and all-cause mortality in patients with COPD. Thorax.

[31] Yue N., Zhang J., Zhao J., Zhang Q., Lin X., Yang J. (2022). Detection and classification of bronchiectasis based on improved Mask-RCNN. Bioeng (Basel).

[32] Chassagnon G., Marini R., Ong V., Da Silva J., Habip Gatenyo D., Honore I. (2025). Identification of structural predictors of lung function improvement in adults with cystic fibrosis treated with elexacaftor-tezacaftor-ivacaftor using deep-learning. Diagn Inter Imaging.

[33] Pusterla O., Heule R., Santini F., Weikert T., Willers C., Andermatt S. (2022). MRI lung lobe segmentation in pediatric cystic fibrosis patients using a recurrent neural network trained with publicly accessible CT datasets. Magn Reson Med.

[34] Ringwald F.G., Wucherpfennig L., Hagen N., Mücke J., Kaletta S., Eichinger M. (2024). Automated lung segmentation on chest MRI in children with cystic fibrosis. Front Med (Lausanne).

[35] Bouzid A.Imene Hadj, Baldacci F., Senneville B.Denis de, Hassen W.Ben, Benlala I., Berger P., Dournes G. (2025). 2025 IEEE 22nd International Symposium on Biomedical Imaging (ISBI). 2025 IEEE 22nd International Symposium on Biomedical Imaging (ISBI).

[36] Longuefosse A., Denis de Senneville B., Dournes G., Benlala I., Baldacci F., Desbarats P. (2025). Anatomical feature-prioritized loss for enhanced MR to CT translation. Phys Med Biol.

[37] Wielpütz M.O., Eichinger M., Biederer J., Wege S., Stahl M., Sommerburg O. (2016). Bildgebung der lunge bei mukoviszidose und klinische interpretation. Rofo.

[38] Graeber S.Y., Sommerburg O., Yu Y., Berges J., Hirtz S., Scheuermann H. (2025). Intestinal current measurement detects age-dependent differences in CFTR function in rectal epithelium. Front Pharm.

[39] Hirtz S., Gonska T., Seydewitz H.H., Thomas J., Greiner P., Kuehr J. (2004). CFTR Cl- channel function in native human colon correlates with the genotype and phenotype in cystic fibrosis. Gastroenterology.

[40] Wielpütz M.O., Eichinger M., Wege S., Eberhardt R., Mall M.A., Kauczor H.-U. (2019). Midterm reproducibility of chest magnetic resonance imaging in adults with clinically stable cystic fibrosis and chronic obstructive pulmonary disease. Am J Respir Crit Care Med.

[41] Ciet P., Serra G., Bertolo S., Spronk S., Ros M., Fraioli F. (2016). Assessment of CF lung disease using motion corrected PROPELLER MRI: a comparison with CT. Eur Radio.

[42] Nyúl L.G., Udupa J.K., Zhang X. (2000). New variants of a method of MRI scale standardization. IEEE Trans Med Imaging.

[43] Tustison N.J., Avants B.B., Cook P.A., Zheng Y., Egan A., Yushkevich P.A., Gee J.C. (2010). N4ITK: improved N3 bias correction. IEEE Trans Med Imaging.

[44] Hagen N., Weichel F., Kühle R., Knaup P., Freudlsperger C., Eisenmann U. (2023). Automated calculation of ontology-based planning proposals: an application in reconstructive oral and maxillofacial surgery. Int J Med Robot.

[45] Mitsuhashi N., Fujieda K., Tamura T., Kawamoto S., Takagi T., Okubo K. (2009). BodyParts3D: 3D structure database for anatomical concepts. Nucleic Acids Res.

[46] Tustison N.J., Qing K., Wang C., Altes T.A., Mugler J.P. (2016). Atlas-based estimation of lung and lobar anatomy in proton MRI. Magn Reson Med.

[47] Martynova, A.; Hagen, N.; Wucherpfennig, L.; Tokur, O.; Ringwald, F.G.; Knaup-Gregori, P. et al., 2024: Lung Lobe Definition on MRI: An Algorithmic Approach. German Medical Science GMS Publishing House. Gesundheit – gemeinsam. Kooperationstagung der Deutschen Gesellschaft für Medizinische Informatik, Biometrie und Epidemiologie (GMDS), Deutschen Gesellschaft für Sozialmedizin und Prävention (DGSMP), Deutschen Gesellschaft für Epidemiologie (DGEpi), Deutschen Gesellschaft für Medizinische Soziologie (DGMS) und der Deutschen Gesellschaft für Public Health (DGPH). DOI: 10.3205/24gmds221.

[48] Simonyan, K.; Zisserman, A., 2014: Very Deep Convolutional Networks for Large-Scale Image Recognition. Available online at 〈http://arxiv.org/pdf/1409.1556v6〉.

[49] Bien N., Rajpurkar P., Ball R.L., Irvin J., Park A., Jones E. (2018). Deep-learning-assisted diagnosis for knee magnetic resonance imaging: development and retrospective validation of MRNet. PLoS Med.

[50] Seeland M., Mäder P. (2021). Multi-view classification with convolutional neural networks. PLoS One.

[51] Selvaraju, R.R.; Cogswell, M.; Das, A.; Vedantam, R.; Parikh, D.; Batra, D., 2016: Grad-CAM: Visual Explanations from Deep Networks via Gradient-based Localization. DOI: 10.48550/arXiv.1610.02391.

[52] Maier-Hein L., Reinke A., Godau P., Tizabi M.D., Buettner F., Christodoulou E. (2024). Metrics reloaded: recommendations for image analysis validation. Nat Methods.

[53] Gwet K.Li (2008). Computing inter-rater reliability and its variance in the presence of high agreement. Br J Math Stat Psychol.

[54] Gwet, Kilem 2012: Handbook of inter-rater reliability: The definitive guide to measuring the extent of agreement among raters.

[55] Virtanen P., Gommers R., Oliphant T.E., Haberland M., Reddy T., Cournapeau D. (2020). SciPy 1.0: fundamental algorithms for scientific computing in python. Nat Methods.

[56] Johnson J.M., Khoshgoftaar T.M. (2019). Survey on deep learning with class imbalance. J Big Data.

[57] Almeida S.D., Norajitra T., Lüth C.T., Wald T., Weru V., Nolden M. (2024). Capturing COPD heterogeneity: anomaly detection and parametric response mapping comparison for phenotyping on chest computed tomography. Front Med (Lausanne).

[58] Huang S.-C., Pareek A., Jensen M., Lungren M.P., Yeung S., Chaudhari A.S. (2023). Self-supervised learning for medical image classification: a systematic review and implementation guidelines. NPJ Digit Med.

[59] Crisosto C., Voskrebenzev A., Gutberlet M., Klimeš F., Kaireit T.F., Pöhler G. (2023). Artificially-generated consolidations and balanced augmentation increase performance of U-net for lung parenchyma segmentation on MR images. PLoS One.

