# Supplementary material for: Automated scoring of airway abnormalities and mucus plugging in chest magnetic resonance imaging of cystic fibrosis using artificial intelligence
Source: Comput Struct Biotechnol J. 2025 Oct 15;28:442–53. doi: 10.1016/j.csbj.2025.10.025 (PMC12593636; doi:10.1016/j.csbj.2025.10.025)
Supplement: Supplementary file 1 — Supplementary material [file mmc1.docx]

# Supplementary material


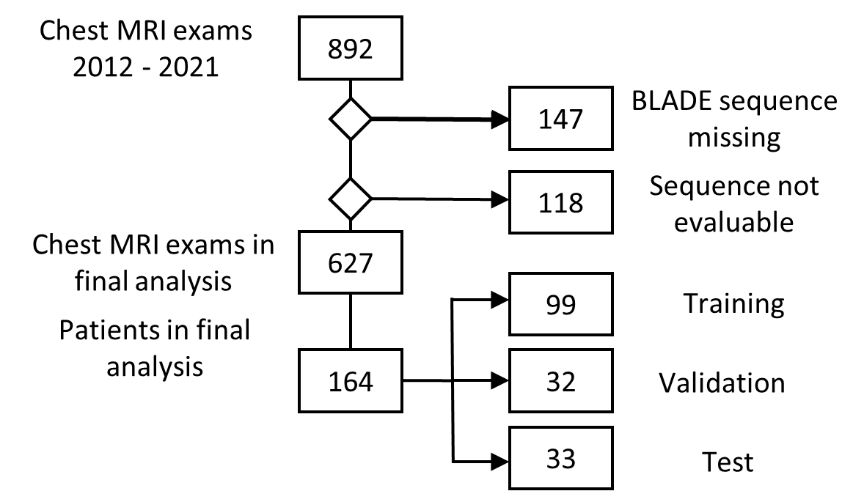


Supplementary Figure 1: Study Flowchart

## Results from model screening

### Score: Bronchiectasis/wall thickening

#### Model: VGG16

|  | Score 0 | Score 1 | Score 2 | Overall |
| --- | --- | --- | --- | --- |
| Accuracy | 0.870 [0.845, 0.891] | 0.809 [0.782, 0.835] | 0.940 [0.922, 0.954] | - |
| Sensitivity | 0.288 [0.212, 0.379] | 0.918 [0.894, 0.937] | 0.706 [0.611, 0.786] | - |
| Specificity | 0.958 [0.941, 0.970] | 0.488 [0.422, 0.555] | 0.972 [0.957, 0.981] | - |
| AUROC | 0.834 [0.790, 0.878] (p=0) | 0.813 [0.664, 0.962] (p=3.79e-05) | 0.972 [0.946, 0.998] (p=0) | Macro: 0.873 |
| CWCE | 0.0383 [0.0306, 0.0670] | 0.0466 [0.0411, 0.0834] | 0.0149 [0.0108, 0.0327] | Macro: 0.0333 |
| Quadratic weighted kappa | - | - | - | 0.564 |
| Gwet’s AC2 | - | - | - | 0.563 |

Supplementary Table 1.1: VGG16 results for bronchiectasis/wall thickening

#### Model: Alexnet

|  | Score 0 | Score 1 | Score 2 | Overall |
| --- | --- | --- | --- | --- |
| Accuracy | 0.866 [0.842, 0.888] | 0.805 [0.777, 0.830] | 0.938 [0.920, 0.953] | - |
| Sensitivity | 0.126 [0.077, 0.201] | 0.957 [0.939, 0.970] | 0.598 [0.501, 0.688] | - |
| Specificity | 0.978 [0.965, 0.987] | 0.352 [0.291, 0.418] | 0.985 [0.974, 0.992] | - |
| AUROC | 0.805 [0.760, 0.850] (p=0) | 0.779 [0.622, 0.935] (p=0.00048) | 0.973 [0.947, 0.999] (p=0) | Macro: 0.852 |
| CWCE | 0.0369 [0.0326, 0.0670] | 0.0423 [0.0364, 0.0766] | 0.0331 [0.0210, 0.0489] | Macro: 0.0375 |
| Quadratic weighted kappa | - | - | - | 0.478 |
| Gwet’s AC2 | - | - | - | 0.475 |

Supplementary Table 1.2: Alexnet results for bronchiectasis/wall thickening

#### Model: Resnet18

|  | Score 0 | Score 1 | Score 2 | Overall |
| --- | --- | --- | --- | --- |
| Accuracy | 0.872 [0.848, 0.893] | 0.736 [0.705, 0.765] | 0.845 [0.819, 0.868] | - |
| Sensitivity | 0.054 [0.025, 0.113] | 0.959 [0.940, 0.972] | 0.020 [0.005, 0.069] | - |
| Specificity | 0.996 [0.988, 0.999] | 0.075 [0.047, 0.119] | 0.958 [0.941, 0.970] | - |
| AUROC | 0.607 [0.542, 0.673] (p=0.00139) | 0.556 [0.378, 0.734] (p=0.537) | 0.265 [0.203, 0.327] (p=9.55e-14) | Macro: 0.476 |
| CWCE | 0.0910 [0.0680, 0.1121] | 0.1046 [0.0892, 0.1427] | 0.1744 [0.1520, 0.1985] | Macro: -0.001 |
| Quadratic weighted kappa | - | - | - | 0.002 |
| Gwet’s AC2 | - | - | - | 0.395 |

Supplementary Table 1.3: Resnet18 results for bronchiectasis/wall thickening

#### ModelResNeXt50_32x4d

|  | Score 0 | Score 1 | Score 2 | Overall |
| --- | --- | --- | --- | --- |
| Accuracy | 0.859 [0.834, 0.881] | 0.735 [0.704, 0.764] | 0.869 [0.844, 0.890] | - |
| Sensitivity | 0.144 [0.091, 0.221] | 0.953 [0.933, 0.967] | 0.000 [0.000, 0.036] | - |
| Specificity | 0.967 [0.952, 0.978] | 0.089 [0.058, 0.135] | 0.988 [0.977, 0.994] | - |
| AUROC | 0.623 [0.556, 0.690] (p=0.000305) | 0.554 [0.372, 0.735] (p=0.564) | 0.311 [0.253, 0.370] (p=2.55e-10) | Macro: 0.496 |
| CWCE | 0.0725 [0.0571, 0.0991] | 0.1019 [0.0836, 0.1363] | 0.1402 [0.1217, 0.1646] | Macro: 0.1048 |
| Quadratic weighted kappa | - | - | - | 0.097 |
| Gwet’s AC2 | - | - | - | 0.096 |

Supplementary Table 1.4: ModelResNeXt50_32x4d results for bronchiectasis/wall thickening

#### Model: EfficientNet-B3

|  | Score 0 | Score 1 | Score 2 | Overall |
| --- | --- | --- | --- | --- |
| Accuracy | 0.869 [0.844, 0.890] | 0.747 [0.716, 0.775] | 0.878 [0.854, 0.898] | - |
| Sensitivity | 0.135 [0.084, 0.211] | 0.964 [0.946, 0.976] | 0.069 [0.034, 0.135] | - |
| Specificity | 0.980 [0.967, 0.988] | 0.103 [0.069, 0.151] | 0.989 [0.979, 0.995] | - |
| AUROC | 0.719 [0.662, 0.775] (p=3.55e-14) | 0.566 [0.381, 0.752] (p=0.484) | 0.608 [0.530, 0.685] (p=0.00651) | Macro: 0.631 |
| CWCE | 0.0652 [0.0477, 0.0877] | 0.1160 [0.0950, 0.1487] | 0.0628 [0.0492, 0.0870] | Macro: 0.0813 |
| Quadratic weighted kappa | - | - | - | 0.170 |
| Gwet’s AC2 | - | - | - | 0.169 |

Supplementary Table 1.5: EfficientNet-B3 results for bronchiectasis/wall thickening

#### Model: DenseNet121

|  | Score 0 | Score 1 | Score 2 | Overall |
| --- | --- | --- | --- | --- |
| Accuracy | 0.862 [0.837, 0.883] | 0.737 [0.707, 0.766] | 0.869 [0.844, 0.890] | - |
| Sensitivity | 0.135 [0.084, 0.211] | 0.954 [0.935, 0.968] | 0.020 [0.005, 0.069] | - |
| Specificity | 0.971 [0.957, 0.981] | 0.094 [0.062, 0.141] | 0.985 [0.974, 0.992] | - |
| AUROC | 0.655 [0.592, 0.718] (p=1.44e-06) | 0.575 [0.395, 0.755] (p=0.416) | 0.157 [0.105, 0.209] (p=0) | 0.462 |
| CWCE | 0.0926 [0.0767, 0.1171] | 0.0956 [0.0750, 0.1276] | 0.1636 [0.1436, 0.1866] | 0.1173 |
| Quadratic weighted kappa | - | - | - | 0.105 |
| Gwet’s AC2 | - | - | - | 0.105 |

Supplementary Table 1.6: DenseNet121 results for bronchiectasis/wall thickening

### Score: Mucus plugging

#### Model: VGG16

|  | Score 0 | Score 1 | Score 2 | Overall |
| --- | --- | --- | --- | --- |
| Accuracy | 0.836 [0.811, 0.858] | 0.818 [0.793, 0.842] | 0.982 [0.972, 0.989] | - |
| Sensitivity | 0.881 [0.854, 0.903] | 0.688 [0.630, 0.741] | 0.636 [0.466, 0.778] | - |
| Specificity | 0.739 [0.687, 0.786] | 0.869 [0.842, 0.892] | 0.995 [0.987, 0.998] | - |
| AUROC | 0.880 [0.783, 0.977] (p=1.35e-14) | 0.846 [0.801, 0.891] (p=0) | 0.983 [0.960, 1.000] (p=0) | Macro: 0.903 |
| CWCE | 0.0919 [0.0752, 0.1154] | 0.0942 [0.0776, 0.1181] | 0.0051 [0.0030, 0.0138] | Macro: 0.0637 |
| Quadratic weighted kappa | - | - | - | 0.685 |
| Gwet’s AC2 | - | - | - | 0.685 |

Supplementary Table 2.1: VGG16 results for bronchiectasis/wall thickening

#### Model: Alexnet

|  | Score 0 | Score 1 | Score 2 | Overall |
| --- | --- | --- | --- | --- |
| Accuracy | 0.831 [0.806, 0.854] | 0.812 [0.786, 0.836] | 0.981 [0.970, 0.988] | - |
| Sensitivity | 0.908 [0.884, 0.928] | 0.613 [0.553, 0.669] | 0.515 [0.352, 0.675] | - |
| Specificity | 0.662 [0.607, 0.713] | 0.889 [0.864, 0.911] | 0.998 [0.992, 0.999] | - |
| AUROC | 0.864 [0.762, 0.966] (p=2.51e-12) | 0.833 [0.785, 0.880] (p=0) | 0.991 [0.971, 1.000] (p=0) | Macro: 0.896 |
| CWCE | 0.0722 [0.0603, 0.0979] | 0.0809 [0.0653, 0.1050] | 0.0104 [0.0050, 0.0188] | Macro: 0.0545 |
| Quadratic weighted kappa | - | - | - | 0.656 |
| Gwet’s AC2 | - | - | - | 0.655 |

Supplementary Table 2.2: Alexnet results for bronchiectasis/wall thickening

#### Model: Resnet18

|  | Score 0 | Score 1 | Score 2 | Overall |
| --- | --- | --- | --- | --- |
| Accuracy | 0.740 [0.711, 0.767] | 0.722 [0.693, 0.749] | 0.965 [0.952, 0.975] | - |
| Sensitivity | 0.804 [0.772, 0.833] | 0.579 [0.519, 0.637] | 0.000 [0.000, 0.104] | - |
| Specificity | 0.599 [0.542, 0.653] | 0.777 [0.745, 0.807] | 1.000 [0.996, 1.000] | - |
| AUROC | 0.752 [0.634, 0.870] (p=2.8e-05) | 0.724 [0.670, 0.778] (p=4.44e-16) | 0.231 [0.169, 0.293] (p=0) | Macro: 0.569 |
| CWCE | 0.1176 [0.0957, 0.1449] | 0.1396 [0.1176, 0.1666] | 0.0370 [0.0290, 0.0508] | Macro: 0.0981 |
| Quadratic weighted kappa | - | - | - | 0.395 |
| Gwet’s AC2 | - | - | - | 0.395 |

Supplementary Table 2.3: Resnet18results for bronchiectasis/wall thickening

#### Model: ResNeXt50_32x4d

|  | Score 0 | Score 1 | Score 2 | Overall |
| --- | --- | --- | --- | --- |
| Accuracy | 0.790 [0.763, 0.815] | 0.758 [0.729, 0.784] | 0.965 [0.952, 0.975] | - |
| Sensitivity | 0.902 [0.877, 0.923] | 0.492 [0.433, 0.552] | 0.000 [0.000, 0.104] | - |
| Specificity | 0.545 [0.488, 0.601] | 0.860 [0.832, 0.884] | 1.000 [0.996, 1.000] | - |
| AUROC | 0.811 [0.700, 0.922] (p=3.74e-08) | 0.756 [0.705, 0.807] (p=0) | 0.577 [0.427, 0.726] (p=0.315) | Macro: 0.715 |
| CWCE | 0.0979 [0.0807, 0.1243] | 0.1308 [0.1105, 0.1578] | 0.0337 [0.0231, 0.0455] | Macro: 0.0875 |
| Quadratic weighted kappa | - | - | - | 0.497 |
| Gwet’s AC2 | - | - | - | 0.490 |

Supplementary Table 2.4: ResNeXt50_32x4d results for bronchiectasis/wall thickening

#### Model: EfficientNet-B3

|  | Score 0 | Score 1 | Score 2 | Overall |
| --- | --- | --- | --- | --- |
| Accuracy | 0.774 [0.747, 0.800] | 0.737 [0.708, 0.764] | 0.958 [0.943, 0.969] | - |
| Sensitivity | 0.820 [0.788, 0.847] | 0.617 [0.557, 0.673] | 0.000 [0.000, 0.104] | - |
| Specificity | 0.676 [0.621, 0.726] | 0.783 [0.751, 0.812] | 0.992 [0.984, 0.996] | - |
| AUROC | 0.826 [0.719, 0.934] (p=2.62e-09) | 0.765 [0.715, 0.816] (p=0) | 0.836 [0.767, 0.905] (p=0) | Macro: 0.809 |
| CWCE | 0.1127 [0.0928, 0.1374] | 0.1258 [0.1063, 0.1536] | 0.0161 [0.0105, 0.0297] | Macro: 0.0849 |
| Quadratic weighted kappa | - | - | - | 0.493 |
| Gwet’s AC2 | - | - | - | 0.493 |

Supplementary Table 2.5: EfficientNet-B3results for bronchiectasis/wall thickening

#### Model: DenseNet121

|  | Score 0 | Score 1 | Score 2 | Overall |
| --- | --- | --- | --- | --- |
| Accuracy | 0.732 [0.703, 0.760] | 0.700 [0.670, 0.728] | 0.965 [0.952, 0.975] | - |
| Sensitivity | 0.766 [0.732, 0.797] | 0.620 [0.561, 0.677] | 0.000 [0.000, 0.104] | - |
| Specificity | 0.659 [0.603, 0.710] | 0.731 [0.696, 0.763] | 1.000 [0.996, 1.000] | - |
| AUROC | 0.752 [0.632, 0.872] (p=3.92e-05) | 0.710 [0.656, 0.765] (p=4.6e-14) | 0.237 [0.174, 0.300] (p=4.44e-16) | Macro: 0.566 |
| CWCE | 0.1802 [0.1527, 0.2052] | 0.1942 [0.1685, 0.2219] | 0.0459 [0.0362, 0.0583] | Macro: 0.1401 |
| Quadratic weighted kappa | - | - | - | 0.425 |
| Gwet’s AC2 | - | - | - | 0.425 |

Supplementary Table 2.6: DenseNet121 results for bronchiectasis/wall thickening

## Lobe wise results for bronchiectasis/wall thickening with Lobe-Expert-Classifiers

### Left upper lobe

|  | Score 0 | Score 1 | Score 2 | Overall |
| --- | --- | --- | --- | --- |
| Accuracy | 0.908 [0.849, 0.945] | 0.894 [0.832, 0.934] | 0.986 [0.950, 0.996] | - |
| Sensitivity | 0.000 [0.000, 0.228] | 0.991 [0.952, 0.998] | 0.933 [0.702, 0.988] | - |
| Specificity | 1.000 [0.971, 1.000] | 0.500 [0.326, 0.674] | 0.992 [0.956, 0.999] | - |
| AUROC | 0.729 [0.585, 0.873] (p=0.00188) | 0.811 [0.251, 1.000] (p=0.277) | 0.991 [0.933, 1.000] (p=0) | Macro: 0.844 |
| CWCE | 0.0664 [0.0550, 0.1279] | 0.0821 [0.0745, 0.1526] | 0.0225 [0.0113, 0.0482] | Macro: 0.0570 |
| Quadratic weighted kappa | - | - | - | 0.648 |
| Gwet’s AC2 | - | - | - | 0.643 |

Supplementary Table 3.1: Lobe-Expert-Classifier left-upper-lobe for bronchiectasis/wall thickening


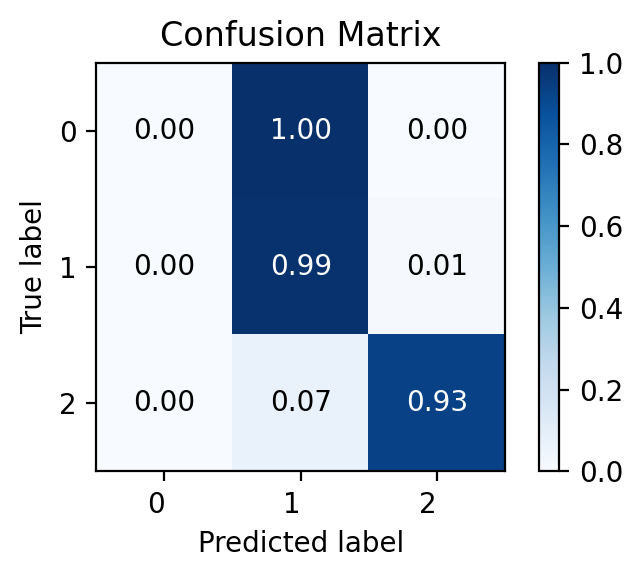


Supplementary Figure 2.1: Normalized confusion matrix for left-upper-lobe prediction of bronchiectasis/wall thickening.

### Lingula

|  | Score 0 | Score 1 | Score 2 | Overall |
| --- | --- | --- | --- | --- |
| Accuracy | 0.752 [0.674, 0.816] | 0.688 [0.607, 0.759] | 0.936 [0.883, 0.966] | - |
| Sensitivity | 0.778 [0.619, 0.883] | 0.677 [0.578, 0.762] | 0.444 [0.189, 0.733] | - |
| Specificity | 0.743 [0.652, 0.817] | 0.711 [0.566, 0.823] | 0.970 [0.925, 0.988] | - |
| AUROC | 0.826 [0.723, 0.930] (p=6.65e-10) | 0.723 [0.439, 1.000] (p=0.124) | 0.926 [0.834, 1.000] (p=0) | Macro: 0.825 |
| CWCE | 0.1156 [0.0969, 0.1982] | 0.1609 [0.1314, 0.2464] | 0.0296 [0.0160, 0.0845] | Macro: 0.102 |
| Quadratic weighted kappa | - | - | - | 0.511 |
| Gwet’s AC2 | - | - | - | 0.503 |

Supplementary Table 3.2: Lobe-Expert-Classifier lingula for bronchiectasis/wall thickening


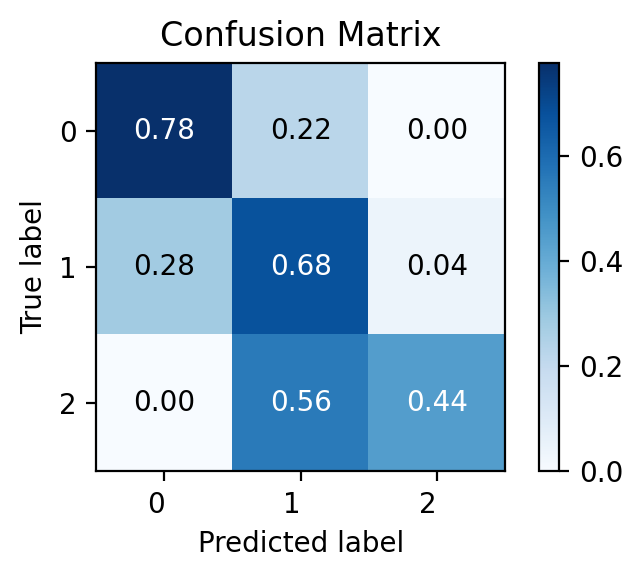


Supplementary Figure 2.2: Normalized confusion matrix for lingula prediction of bronchiectasis/wall thickening.

### Left lower lobe

|  | Score 0 | Score 1 | Score 2 | Overall |
| --- | --- | --- | --- | --- |
| Accuracy | 0.858 [0.791, 0.906] | 0.773 [0.697, 0.834] | 0.915 [0.857, 0.951] | - |
| Sensitivity | 0.000 [0.000, 0.161] | 0.991 [0.950, 0.998] | 0.000 [0.000, 0.259] | - |
| Specificity | 1.000 [0.969, 1.000] | 0.000 [0.000, 0.110] | 0.992 [0.958, 0.999] | - |
| AUROC | 0.835 [0.738, 0.932] (p=1.48e-11) | 0.686 [0.229, 1.000] (p=0.424) | 0.945 [0.872, 1.000] (p=0) | Macro: 0.822 |
| CWCE | 0.0831 [0.0692, 0.1519] | 0.0859 [0.0915, 0.1907] | 0.0556 [0.0280, 0.1022] | Macro: 0.0749 |
| Quadratic weighted kappa | - | - | - | 0.004 |
| Gwet’s AC2 | - | - | - | -0.007 |

Supplementary Table 3.3: Lobe-Expert-Classifier left-lower-lobe for bronchiectasis/wall thickening


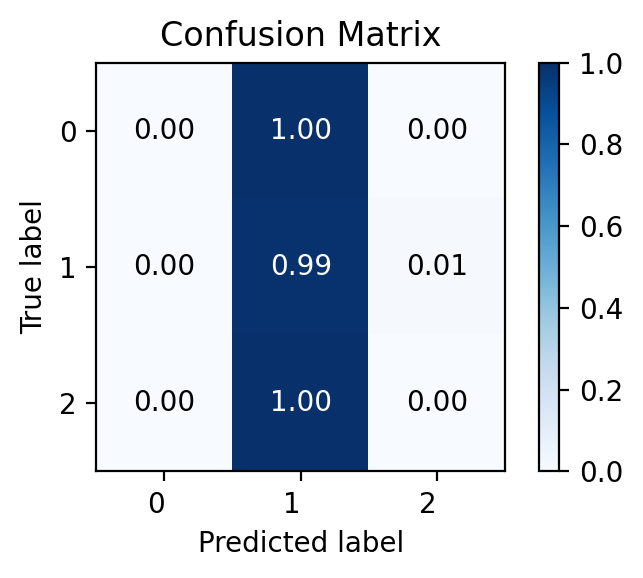


Supplementary Figure 2.3: Normalized confusion matrix for left-lower-lobe prediction of bronchiectasis/wall thickening.

### Right upper lobe

|  | Score 0 | Score 1 | Score 2 | Overall |
| --- | --- | --- | --- | --- |
| Accuracy | 0.936 [0.883, 0.966] | 0.887 [0.824, 0.929] | 0.950 [0.901, 0.976] | - |
| Sensitivity | 0.000 [0.000, 0.299] | 0.952 [0.892, 0.979] | 0.929 [0.774, 0.980] | - |
| Specificity | 1.000 [0.972, 1.000] | 0.703 [0.542, 0.825] | 0.956 [0.901, 0.981] | - |
| AUROC | 0.709 [0.611, 0.807] (p=2.9e-05) | 0.886 [0.573, 1.000] (p=0.0157) | 0.986 [0.922, 1.000] (p=0) | Macro: 0.861 |
| CWCE | 0.0650 [0.0431, 0.1116] | 0.0916 [0.0654, 0.1512] | 0.0442 [0.0161, 0.0781] | Macro: 0.0669 |
| Quadratic weighted kappa | - | - | - | 0.732 |
| Gwet’s AC2 | - | - | - | 0.729 |

Supplementary Table 3.4: Lobe-Expert-Classifier right-upper-lobe for bronchiectasis/wall thickening


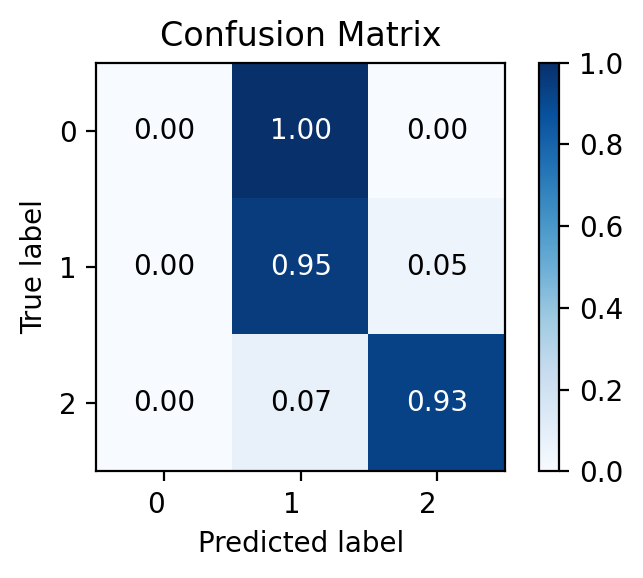


Supplementary Figure 2.4: Normalized confusion matrix for right-upper-lobe prediction of bronchiectasis/wall thickening.

### Right middle lobe

|  | Score 0 | Score 1 | Score 2 | Overall |
| --- | --- | --- | --- | --- |
| Accuracy | 0.901 [0.840, 0.940] | 0.830 [0.759, 0.883] | 0.929 [0.874, 0.961] | - |
| Sensitivity | 0.000 [0.000, 0.215] | 0.971 [0.919, 0.990] | 0.696 [0.491, 0.844] | - |
| Specificity | 1.000 [0.971, 1.000] | 0.432 [0.287, 0.591] | 0.975 [0.928, 0.991] | - |
| AUROC | 0.782 [0.638, 0.926] (p=0.000122) | 0.786 [0.398, 1.000] (p=0.149) | 0.973 [0.908, 1.000] (p=0) | Macro: 0.847 |
| CWCE | 0.1006 [0.0729, 0.1446] | 0.0742 [0.0938, 0.1838] | 0.0420 [0.0226, 0.0877] | Macro: 0.0723 |
| Quadratic weighted kappa | - | - | - | 0.552 |
| Gwet’s AC2 | - | - | - | 0.549 |

Supplementary Table 3.5: Lobe-Expert-Classifier right-middle-lobe for bronchiectasis/wall thickening


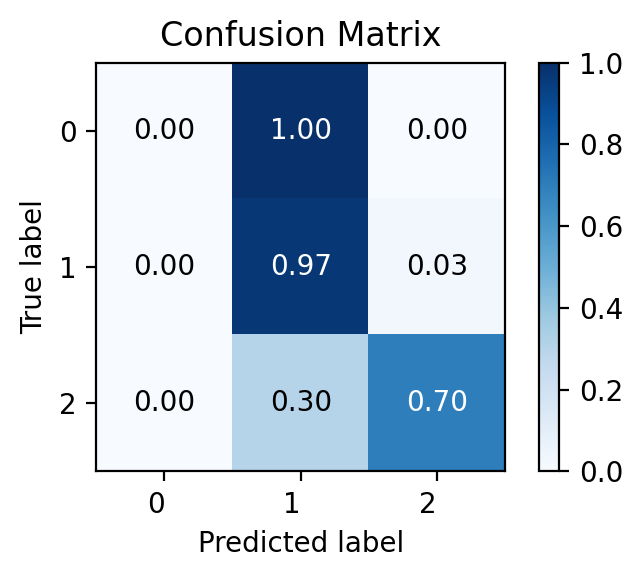


Supplementary Figure 2.5: Normalized confusion matrix for right-middle-lobe prediction of bronchiectasis/wall thickening.

### Right lower lobe

|  | Score 0 | Score 1 | Score 2 | Overall |
| --- | --- | --- | --- | --- |
| Accuracy | 0.865 [0.799, 0.912] | 0.801 [0.728, 0.859] | 0.936 [0.883, 0.966] | - |
| Sensitivity | 0.100 [0.028, 0.301] | 0.982 [0.936, 0.995] | 0.273 [0.097, 0.566] | - |
| Specificity | 0.992 [0.955, 0.999] | 0.161 [0.071, 0.326] | 0.992 [0.958, 0.999] | - |
| AUROC | 0.883 [0.794, 0.971] (p=0) | 0.804 [0.383, 1.000] (p=0.157) | 0.962 [0.898, 1.000] (p=0) | Macro: 0.883 |
| CWCE | 0.0749 [0.0567, 0.1306] | 0.0729 [0.0822, 0.1744] | 0.0412 [0.0159, 0.0758] | Macro: 0.0630 |
| Quadratic weighted kappa | - | - | - | 0.266 |
| Gwet’s AC2 | - | - | - | 0.259 |

Supplementary Table 3.6: Lobe-Expert-Classifier right-lower-lobe for bronchiectasis/wall thickening


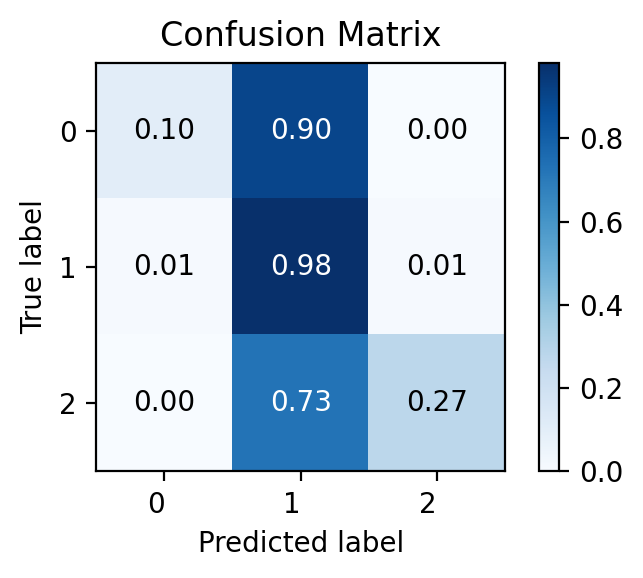


Supplementary Figure 2.6: Normalized confusion matrix for right-lower-lobe prediction of bronchiectasis/wall thickening.

|  | Value | 95% CI |
| --- | --- | --- |
| ICC(2,1) | 0.640 | 0.610–0.830 |
| Pearson r | 0.710* | 0.680–0.832* |

Supplementary Table 3.7: Results of overall lobe scoring (0-12) for all combined Lobe-Expert-Classifiers for bronchiectasis/wall thickening

## Lobe wise results for mucus plugging with Lobe-Expert-Classifiers

### Left upper lobe

|  | Score 0 | Score 1 | Score 2 | Overall |
| --- | --- | --- | --- | --- |
| Accuracy | 0.791 [0.723, 0.847] | 0.779 [0.709, 0.836] | 0.988 [0.956, 0.997] | - |
| Sensitivity | 0.966 [0.915, 0.987] | 0.302 [0.186, 0.451] | 0.500 [0.150, 0.850] | - |
| Specificity | 0.362 [0.240, 0.505] | 0.950 [0.895, 0.977] | 1.000 [0.976, 1.000] | - |
| AUROC | 0.833 [0.554, 1.000] (p=0.0195) | 0.803 [0.688, 0.918] (p=2.33e-07) | 0.992 [0.942, 1.000] (p=0) | Macro: 0.876 |
| CWCE | 0.0938 [0.0889, 0.1761] | 0.0996 [0.0902, 0.1846] | 0.0157 [0.0044, 0.0332] | Macro: 0.0697 |
| Quadratic weighted kappa | - | - | - | 0.497 |
| Gwet’s AC2 | - | - | - | 0.48 |

Supplementary Table 4.1: Lobe-Expert-Classifier left-upper-lobe for mucus plugging


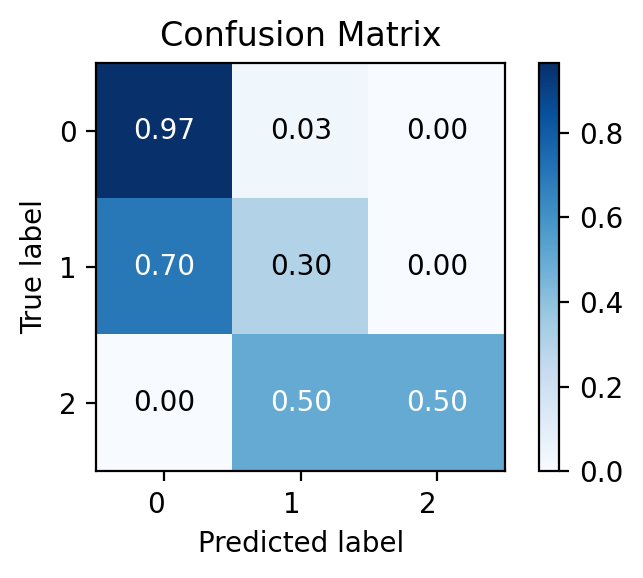


Supplementary Figure 3.1: Normalized confusion matrix for left-upper-lobe prediction of mucus plugging.

### Lingula

|  | Score 0 | Score 1 | Score 2 | Overall |
| --- | --- | --- | --- | --- |
| Accuracy | 0.804 [0.736, 0.857] | 0.791 [0.723, 0.847] | 0.988 [0.956, 0.997] | - |
| Sensitivity | 0.991 [0.953, 0.998] | 0.279 [0.167, 0.427] | 0.500 [0.150, 0.850] | - |
| Specificity | 0.340 [0.222, 0.483] | 0.975 [0.929, 0.991] | 1.000 [0.976, 1.000] | - |
| AUROC | 0.828 [0.538, 1.000] (p=0.0267) | 0.790 [0.671, 0.909] (p=1.89e-06) | 0.995 [0.947, 1.000] (p=0) | Macro: 0.871 |
| CWCE | 0.0842 [0.0953, 0.1819] | 0.0847 [0.0883, 0.1815] | 0.0176 [0.0055, 0.0377] | Macro: 0.0622 |
| Quadratic weighted kappa | - | - | - | 0.515 |
| Gwet’s AC2 | - | - | - | 0.492 |

Supplementary Table 4.2: Lobe-Expert-Classifier lingula for mucus plugging


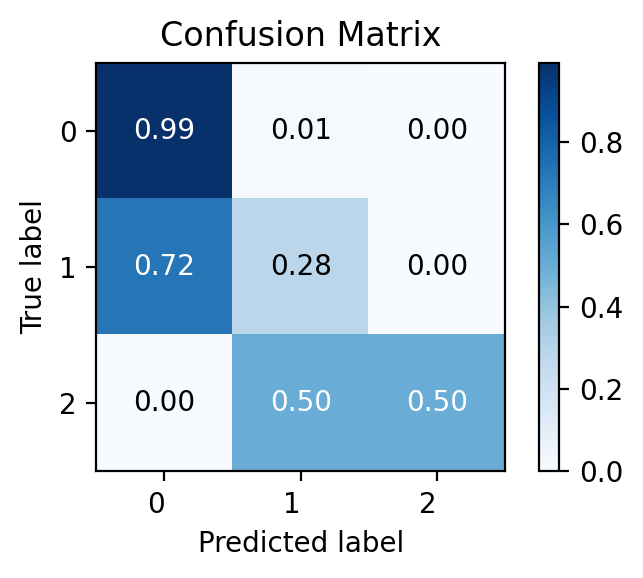


Supplementary Figure 3.2: Normalized confusion matrix for lingula prediction of mucus plugging.

### Left lower lobe

|  | Score 0 | Score 1 | Score 2 | Overall |
| --- | --- | --- | --- | --- |
| Accuracy | 0.680 [0.607, 0.746] | 0.675 [0.601, 0.741] | 0.994 [0.967, 0.999] | - |
| Sensitivity | 0.879 [0.796, 0.931] | 0.429 [0.324, 0.540] | 1.000 [0.207, 1.000] | - |
| Specificity | 0.449 [0.343, 0.559] | 0.880 [0.798, 0.932] | 0.994 [0.967, 0.999] | - |
| AUROC | 0.760 [0.590, 0.931] (p=0.00277) | 0.754 [0.609, 0.900] (p=0.000607) | nan [nan, nan] (p=nan) | Macro: 0.838 |
| CWCE | 0.1731 [0.1322, 0.2471] | 0.1680 [0.1342, 0.2496] | 0.0055 [0.0010, 0.0151] | Macro: 0.1155 |
| Quadratic weighted kappa | - | - | - | 0.376 |
| Gwet’s AC2 | - | - | - | 0.355 |

Supplementary Table 4.3: Lobe-Expert-Classifier left-lower-lobe for mucus plugging


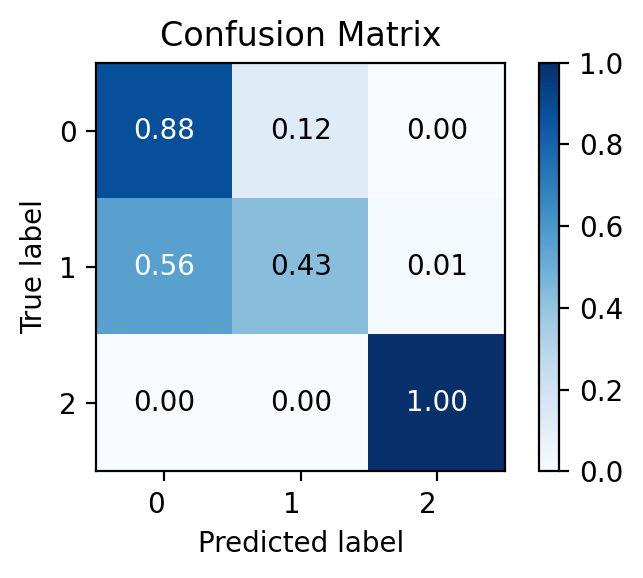


Supplementary Figure 3.3: Normalized confusion matrix for left-lower-lobe prediction of mucus plugging.

### Right upper lobe

|  | Score 0 | Score 1 | Score 2 | Overall |
| --- | --- | --- | --- | --- |
| Accuracy | 0.722 [0.650, 0.784] | 0.669 [0.595, 0.735] | 0.935 [0.887, 0.963] | - |
| Sensitivity | 0.966 [0.905, 0.988] | 0.386 [0.280, 0.503] | 0.000 [0.000, 0.259] | - |
| Specificity | 0.457 [0.353, 0.565] | 0.869 [0.788, 0.922] | 1.000 [0.976, 1.000] | - |
| AUROC | 0.812 [0.657, 0.967] (p=8.29e-05) | 0.721 [0.579, 0.863] (p=0.00224) | 0.959 [0.904, 1.000] (p=0) | Macro: 0.831 |
| CWCE | 0.1525 [0.1211, 0.2189] | 0.1320 [0.1126, 0.2207] | 0.0492 [0.0204, 0.0860] | Macro: 0.1113 |
| Quadratic weighted kappa | - | - | - | 0.457 |
| Gwet’s AC2 | - | - | - | 0.414 |

Supplementary Table 4.4: Lobe-Expert-Classifier right-upper-lobe for mucus plugging


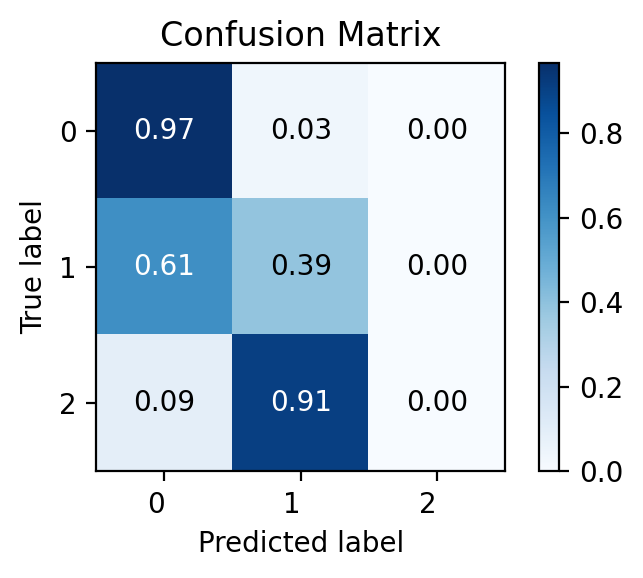


Supplementary Figure 3.4: Normalized confusion matrix for right-upper-lobe prediction of mucus plugging.

### Right middle lobe

|  | Score 0 | Score 1 | Score 2 | Overall |
| --- | --- | --- | --- | --- |
| Accuracy | 0.781 [0.713, 0.837] | 0.692 [0.619, 0.757] | 0.899 [0.845, 0.936] | - |
| Sensitivity | 0.855 [0.753, 0.919] | 0.632 [0.519, 0.731] | 0.375 [0.212, 0.573] | - |
| Specificity | 0.730 [0.636, 0.807] | 0.742 [0.645, 0.820] | 0.986 [0.951, 0.996] | - |
| AUROC | 0.872 [0.759, 0.984] (p=8.9e-11) | 0.767 [0.626, 0.908] (p=0.000199) | 0.970 [0.912, 1.000] (p=0) | Macro: 0.870 |
| CWCE | 0.0717 [0.0707, 0.1585] | 0.1350 [0.1124, 0.2226] | 0.0726 [0.0393, 0.1144] | Macro: 0.0931 |
| Quadratic weighted kappa | - | - | - | 0.627 |
| Gwet’s AC2 | - | - | - | 0.620 |

Supplementary Table 4.5: Lobe-Expert-Classifier right-middle-lobe for mucus plugging


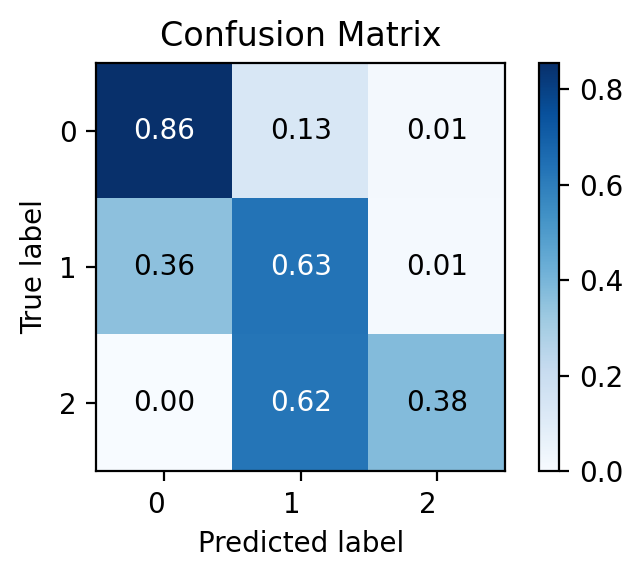


Supplementary Figure 3.5: Normalized confusion matrix for right-middle-lobe prediction of mucus plugging.

### Right lower lobe

|  | Score 0 | Score 1 | Score 2 | Overall |
| --- | --- | --- | --- | --- |
| Accuracy | 0.775 [0.706, 0.832] | 0.757 [0.688, 0.816] | 0.982 [0.949, 0.994] | - |
| Sensitivity | 0.938 [0.870, 0.971] | 0.535 [0.420, 0.646] | 0.000 [0.000, 0.658] | - |
| Specificity | 0.562 [0.448, 0.670] | 0.918 [0.847, 0.958] | 0.994 [0.967, 0.999] | - |
| AUROC | 0.799 [0.620, 0.978] (p=0.00108) | 0.779 [0.643, 0.916] (p=6.22e-05) | 0.949 [0.845, 1.000] (p=0) | Macro: 0.842 |
| CWCE | 0.0968 [0.0855, 0.1847] | 0.0949 [0.0896, 0.1916] | 0.0076 [0.0033, 0.0293] | Macro: 0.0664 |
| Quadratic weighted kappa | - | - | - | 0.525 |
| Gwet’s AC2 | - | - | - | 0.513 |

Supplementary Table 4.6: Lobe-Expert-Classifier right-lower-lobe for mucus plugging


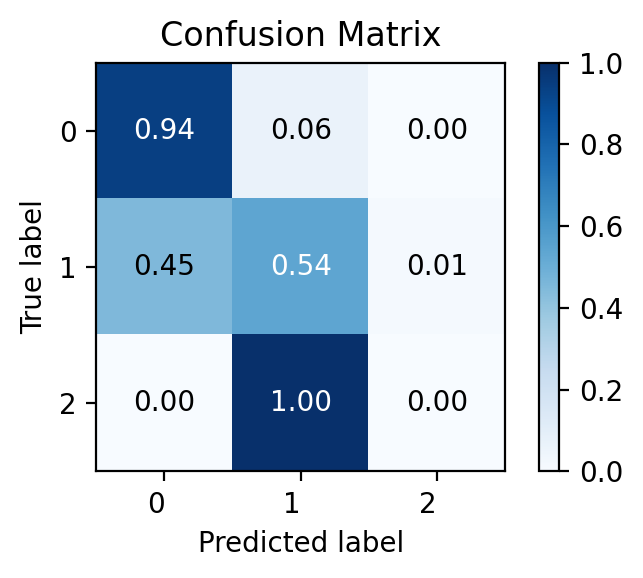


Supplementary Figure 3.6: Normalized confusion matrix for right-lower-lobe prediction of mucus plugging.

|  | Value | 95% CI |
| --- | --- | --- |
| ICC(2,1) | 0.542 | 0.424–0.653 |
| Pearson r | 0.648* | 0.535–0.747 |

Supplementary Table 4.7: Results of overall lobe scoring (0-12) for all combined Lobe-Expert-Classifiers for mucus plugging
